# Sex-Specific Associations of Testosterone and Genetic Factors With Health Span

**DOI:** 10.3389/fendo.2021.773464

**Published:** 2021-11-25

**Authors:** Xiaoyu Zhao, Shuang Liang, Nanxi Wang, Tongtong Hong, Muhammed Lamin Sambou, Jingyi Fan, Meng Zhu, Cheng Wang, Dong Hang, Yue Jiang, Juncheng Dai

**Affiliations:** ^1^ Department of Epidemiology, Center for Global Health, School of Public Health, Nanjing Medical University, Nanjing, China; ^2^ Jiangsu Key Lab of Cancer Biomarkers, Prevention and Treatment, Collaborative Innovation Center for Cancer Personalized Medicine and China International Cooperation Center for Environment and Human Health, Gusu School, Nanjing Medical University, Nanjing, China

**Keywords:** health span, testosterone, polygenic risk score, sex-specific, UK Biobank

## Abstract

**Background:**

Previous studies have suggested associations between testosterone, genetic factors, and a series of complex diseases, but the associations with the lifespan phenotype, such as health span, remain unclear.

**Methods:**

In this prospective cohort study, we analyzed 145,481 men and 147,733 women aged 38–73 years old from UK Biobank (UKB) to investigate the sex-specific associations of total testosterone (TT), free testosterone (FT), or polygenic risk score (PRS) with health span termination (HST) risk. At baseline, serum testosterone levels were measured. HST was defined by eight events strongly associated with longevity. PRS, an efficient tool combining the effect of common genetic variants to discriminate genetic risk of complex phenotypes, was constructed by 12 single-nucleotide polymorphisms related to health span from UKB (*P* ≤ 5.0 × 10^−8^). We used multivariable Cox regression models to estimate hazard ratios (HRs) and 95% confidence intervals (CIs).

**Results:**

With a median follow-up time of 7.70 years, 26,748 (18.39%) men and 18,963 (12.84%) women had HST. TT was negatively associated with HST in men [HR per standard deviation (SD) increment of log-TT: 0.92, 95% CI: 0.88–0.97]. Inversely, both TT (HR per SD increment of log-TT: 1.05, 95% CI: 1.02–1.08) and FT (HR per SD increment of log-FT: 1.08, 95% CI: 1.05–1.11) presented an increased risk of HST in women. PRS was positively associated with HST risk (quintile 5 *versus* quintile 1, men, HR: 1.19, 95% CI: 1.15–1.24; women, HR: 1.21, 95% CI: 1.16–1.27). Moreover, men with high TT and low genetic risk showed the lowest HST risk (HR: 0.80, 95% CI: 0.73–0.88), whereas HST risk for women with both high TT and genetic risk increased obviously (HR: 1.32, 95% CI: 1.19–1.46). Similar joint effects were observed for FT in both genders.

**Conclusions:**

We observed sex-specific associations that testosterone was negatively associated with HST risk in men and positively associated with HST risk in women. Genetic factors increased the HST risk, suggesting that participants with both high genetic risk and abnormal testosterone levels (high level in women or low level in men) should be the target for early intervention. Although our findings highlight the associations between testosterone and health span, further mechanistic studies and prospective trials are warranted to explore the causation behind.

## Introduction

Aging is becoming one of the most substantial social transformations of developed countries in the twenty-first century. Although the average life expectancy in most developed countries has nearly doubled over the past two centuries (1), the increase of health span is more significant and meaningful than simply living longer. Briefly, “health span” is a period of good health during the lifetime and is also termed as healthy and morbidity-free lifespan (2). In many supercentenarians, health span approximates lifespan due to a consistent delay in the occurrence of age-related diseases (3), which has stimulated enormous interest in mysteries behind. Hence, health span becomes a promising phenotype for longevity research.

Interestingly, men usually have a shorter life expectancy than women, suggesting men seem to age slightly faster than women (4). Considering the important effect of sex hormones on maintaining individual muscle mass, bone mass, and physical function, it is rational to speculate that sex hormones have a potential relationship with individual aging. Testosterone is a sex hormone mainly produced by testes in men or synthesized by ovaries and adrenal glands of women in small amounts. Majority of testosterone circulating in the blood is tightly bound to sex hormone-binding globulin (SHBG) or weakly bound to albumin. Approximately only 1%–3% of them is unbound and readily available to the tissues, referred to as free testosterone (FT) (5). It is worth noting that the concentrations of serum testosterone and free testosterone decrease with age in both genders (6), and deficiencies in multiple anabolic hormones have been shown to associate with individual longevity (7). Previous studies have revealed the associations between testosterone and several complex diseases, such as cardiovascular diseases (8), metabolic disease (9), and cancer (10, 11), as well as mortality (12, 13). However, direct evaluation on the effect of testosterone for health span is limited. Additionally, given sex differences in serum testosterone concentrations and biological mechanisms, sex-specific analyses for health span are also necessary.

Recently, a genome-wide association study (GWAS) systematically selected eight health events closely connected with aging to construct a reasonable definition for health span, and further identified 12 single-nucleotide polymorphisms (SNPs) related to health span (14). This provides a great chance to establish polygenic risk score (PRS) for health span. To our knowledge, PRS is an efficient tool estimating the cumulative effect of multiple risk-associated variants to discriminate populations at high risk of complex diseases (15), such as lung cancer (16) and breast cancer (17). Considering that an interplay between serum testosterone and genetic factors may exist, investigation for their joint association with health span is essential as well.

Therefore, based on complete clinical and genetic data from UK Biobank (UKB), we explored the sex-specific associations between endogenous testosterone, genetic factors, and health span by a large-scale sample including 293,214 participants.

## Materials and Methods

### Study Participants

The UK Biobank is an ongoing national prospective cohort study with a detailed protocol publicly available (18). In brief, nearly 500,000 volunteers aged 40 to 70 years were enrolled from 22 assessment centers across England, Wales, and Scotland between 2006 and 2010. At baseline, characteristics of participants are assessed by a self-administered questionnaire, physical assessments, brief interview, and blood collection. The UK Biobank study has approval by the North West Multi-Centre Research Ethics Committee (http://www.ukbiobank.ac.uk/ethics/).

In this study, we successively excluded 72,477 and 29,027 participants whose health span had terminated at baseline according to in-patient hospital admissions data (UKB category 2000) and self-reported diagnoses obtained *via* verbal interview (UKB category 100074). Additionally, 23,721 non-white participants and 84,068 participants with missing data on testosterone, SHBG, or albumin were both excluded. Finally, a total of 145,481 men and 147,733 women were included for the following analyses (**Supplementary Figure 1**).

### Assessment of Biomarkers

Details about serum biomarker measurements have been described elsewhere (19, 20). In short, serum samples were prepared and stored at −80°C until assayed for testosterone, albumin, and SHBG in the UK Biobank central laboratory. Then, serum concentrations of TT and SHBG were measured on the platform of Beckman Coulter DXI 800 by chemiluminescent immunoassays, with reportable ranges of 0.35–55.52 and 0.33–242 nmol/L, respectively. Albumin was assayed on Beckman Coulter AU5800 by a colorimetric method, with a reportable range of 15–60 g/L. The coefficients of variation of internal quality control samples with known high, medium, and low concentrations for TT, SHBG, and albumin ranged from 3.66% to 8.34%, 5.22% to 5.67%, and 2.09% to 2.20%, respectively. Moreover, the assay of serum biomarkers was registered with an external quality assurance scheme to verify the accuracy of measurement, and the high proportions of participated distributions for TT (99%), SHBG (95%), and albumin (97%) showed good or acceptable assay performance. FT concentrations were calculated from assayed values of TT, SHBG, albumin, and the association constants for the binding of testosterone to SHBG and albumin by using the Vermeulen equation (21).

### Ascertainment of Outcome

Following the law of Gompertz dynamics (22), Zenin et al. identified a cluster of the top 8 morbidities with an independent clinical manifestation to define health span. These events included congestive heart failure (CHF), myocardial infarction (MI), chronic obstructive pulmonary disease (COPD), stroke, dementia, diabetes, dancer, and death (14). In this study, participants that had the first experience of any of these events mentioned above during the follow-up were considered to have “health span termination (HST)”, which marked a shift into a biological or clinical phase with a hazard of frailty, multimorbidity, and premature death accumulated progressively.

We extracted data from the National Cancer Registry (UKB data category 100092) and National Death Registry (UKB data category 100093) to define cancer and death events and followed a list of hospital data codes and self-reported data codes to define the other six events (**Supplementary Table 1**). To ensure consistency of updated time between these three databases, we set “December 14, 2016” as the end of follow-up. We calculated follow-up time from the date of attending an assessment center until the date of HST or “December 14, 2016”, whichever occurred first.

### Polygenic Risk Score Calculation

A detailed procedure for genetic data in UKB has been reported elsewhere (23). We included 12 SNPs that were genome-wide significantly (*P* ≤ 5.0 × 10^−8^) associated with health span in Caucasians (**Supplementary Table 2**) (14). PRS was created using a weighted strategy under an additive model as follows:


$$PRS=\beta_{1}\times SNP_{i,1}+\beta_{2}\times SNP_{i,2}+\cdots +\beta_{j}\times SNP_{i,j}$$

Briefly, the dosage of risk allele for each individual (*SNP_ij_*) was summed after weighting with its respective effect size of the site-specific phenotype (*ß_j_*).

### Statistical Analyses

Cox proportional hazards regression models were used to estimate hazard ratios (HRs) and 95% confidence intervals (CIs) of the associations between testosterone and HST risk, with adjustment for age, menopause status (women only), Townsend deprivation index, education, body mass index (BMI), smoking status, alcohol intake frequency, physical activity, healthy diet (24), family history of cancer and cardiac-cerebral vascular disease (CCVD), aspirin/ibuprofen use, and hormone replacement therapy use (HRT, women only). To clarify the independent effect of TT, sex hormone-binding globulin (SHBG) was additionally adjusted in the fully adjusted model of TT (see **Supplementary Methods** for details) (12, 13). We also explored the linear association of testosterone after natural log transformation. The proportional hazard assumption was assessed based on Schoenfeld residuals.

Then, we visualized the concrete associations between testosterone and risk of HST by multivariate cubic regression splines with 4 knots for further exploration. Likelihood ratio was adopted to test potential non-linearity by comparing the model with only a linear term against the model with both linear and cubic spline terms. We also separately assessed the association of testosterone with cause-specific incidence [death, cancer (colorectal, lung, prostate, breast, ovary and endometrial cancer), congestive heart failure, chronic obstructive pulmonary disease, myocardial infarction, dementia, diabetes, and stroke] for better understanding the internal association with health span. Moreover, joint effects of TT and SHBG were also evaluated because of potential biological interplay, and stratification analyses were conducted to explore the heterogeneity of associations across the subgroups.

Models with and without the interaction terms between health span PRS and testosterone were compared using likelihood ratio tests to evaluate the statistical significance of potential effect modification. Participants were categorized into low (the bottom quintile, Q1), moderate (the second to fourth quintiles, Q2–Q4), and high (the top quintile, Q5) genetic strata. A Cox regression model, additionally adjusted for the top 10 genetic principal components (PC1–10) and genotyping chip, was executed to examine the risk of HST among those with a high or intermediate genetic risk, compared with participants with low genetic risk. Furthermore, combined analyses of the risk of HST were performed across different genetic risk and TT/FT categories. Finally, sensitivity analyses were performed to test the robustness of the observed associations by excluding participants who had HST in the first 2 years or with poor self-reported health status at baseline, as well as those with outliers of testosterone (top 1% and bottom 1%). We also additionally adjusted the fasting time and menstrual cycle proxy variables (“Time since last menstrual period”, “Length of menstrual cycle”, and “Menstruating today”) to control the potential bias introduced by these variables.

All analyses were performed in R (version 3.6.3). Cox regression and multivariate cubic regression splines were performed by “survival” and “rms” R packages. *P <*0.05 was statistically significant.

## Results

Totally, the median follow-up time was 7.70 years (interquartile range: 6.80–8.45 years). Nearly half of the HST events were caused by cancer (46.3%), followed by MI (17.8%) and death (11.4%) (**Supplementary Table 3**).

The baseline characteristics of the participants by sex and health span are described in **Table 1**. There were 26,748 (18.39%) men and 18,963 (12.84%) women with HST during the follow-up. Men with HST were older and had a lower TT and FT level. They were more likely to have higher BMI and Townsend deprivation index. Moreover, they also tended to be current smokers and have a family history of CCVD or cancer. Similar characteristics were observed in women with HST. The majority of these women had reached menopause, were hormone replacement therapy users, and had higher levels of FT than the healthy women. Additionally, characteristics of participants by quintiles of total testosterone were also performed (**Supplementary Table 4**). The non-normality distribution of TT and FT was exhibited clearly, and the levels of TT and FT were totally different in both genders (**Supplementary Figure 2**).

**Table 1:** Baseline characteristics of participants with or without terminated health span.

Characteristics	Men (*n* = 145,481)		Women (*n* = 147,733)	
	Unterminated (*n* = 118,733)	Terminated (*n* = 26,748)	Unterminated (*n* = 128,770)	Terminated (*n* = 18,963)
TT, mean (SD), nmol/L	12.2 (3.6)	12.0 (3.7)	1.1 (0.7)	1.1 (0.7)
FT, mean (SD), pmol/L	217.6 (61.9)	205.2 (59.4)	14.5 (10.7)	15.2 (11.3)
Age, mean (SD), years	55.0 (8.1)	60.1 (6.7)	55.1 (8.0)	58.9 (7.4)
TDI, mean (SD)	−1.5 (3.0)	−1.4 (3.1)	−1.6 (2.9)	−1.4 (3.0)
BMI, mean (SD), kg/m^2^	27.5 (3.9)	28.0 (4.3)	26.8 (4.9)	27.8 (5.5)
Had college degree (%)	43,466 (36.6)	7,598 (28.4)	42,724 (33.2)	5,066 (26.7)
Smoking status (%)				
Never	62,862 (52.9)	11,193 (41.9)	78,593 (61.0)	10,375 (54.7)
Previous	41,961 (35.3)	11,227 (42.0)	39,165 (30.4)	6,192 (32.7)
Current	13,593 (11.5)	4,216 (15.8)	10,641 (8.3)	2,310 (12.2)
Alcohol intake (%)				
Heavy	64,480 (54.3)	14,544 (54.4)	51,391 (39.9)	6,907 (36.5)
Moderate	42,174 (35.5)	8,852 (33.1)	51,986 (40.4)	7,271 (38.4)
Light	12,007 (10.1)	3,320 (12.4)	25,318 (19.7)	4,772 (25.2)
IPAQ activity group (%)				
High	45,129 (38.0)	9,520 (35.6)	39,708 (30.8)	5,355 (28.2)
Moderate	38,816 (32.7)	8,601 (32.2)	44,153 (34.3)	6,208 (32.7)
Low	18,003 (15.2)	4,222 (15.8)	18,018 (14.0)	2,801 (14.8)
Healthy diet (%)	78,525 (66.1)	17,738 (66.3)	105,740 (82.1)	15,379 (81.1)
Family history (%)				
CCVD	61,270 (51.6)	15,296 (57.2)	73,508 (57.1)	11,803 (62.2)
Cancer	40,659 (34.2)	10,172 (38.0)	44,824 (34.8)	7,308 (38.5)
Aspirin/ibuprofen use (%)	25,704 (21.7)	7,075 (26.5)	30,444 (23.6)	4,738 (25.0)
Ever used HRT (%)	–	–	42,808 (33.2)	8,640 (45.6)
Had menopause (%)	–	–	73,446 (57.0)	13,215 (69.7)

The Kruskal–Wallis one-way ANOVA test for continuous variables and the chi-squared test for categorical variables were used to calculate the P-values across men and women with or without terminated health span, respectively, and the variables listed all had a P-value <0.05. Data presented as mean (SD) or numbers (%).

TT, total testosterone; FT, free testosterone; TDI, Townsend deprivation index; BMI, body mass index; SD, standard deviation; IPAQ, International Physical Activity Questionnaire; CCVD, cardiac-cerebral vascular disease; HRT, hormone replacement therapy.

The sex-specific associations between TT, FT, and risk of HST are exhibited in **Table 2**. For men, those in the second to fourth quintiles (Q2–Q4) of TT all had a lower HST risk in the fully adjusted models with the bottom quintile (Q1) of TT as a reference (Q2, HR: 0.94, 95% CI: 0.91–0.98; Q3, HR: 0.95, 95% CI: 0.91–0.99; Q4, HR: 0.94, 95% CI: 0.90–0.98), and the hazard decreased by 8% per SD (standard deviation) increment of log-TT (HR: 0.92, 95% CI: 0.88–0.97). However, the association of FT in men diminished in the fully adjusted model. Compared with women in the bottom quintile (Q1), only those in the top quintile (Q5) of TT (HR: 1.08, 95% CI: 1.04–1.13) and FT (HR: 1.12, 95% CI: 1.07–1.18) had a significantly increased HST risk.

**Table 2:** Associations of total testosterone and free testosterone with risk of health span termination in both genders.

	Hazard ratio (95% CI)					*P* for trend	HR per log SD increase
	Quintile 1^a^	Quintile 2	Quintile 3	Quintile 4	Quintile 5		
**Men (*n* = 145,481)**							
**Total testosterone**							
No. of events (%)	5,988 (20.6)	5,354 (18.4)	5,255 (18.1)	5,085 (17.5)	5,066 (17.4)		
Basic model^b^	Ref	0.90 (0.87–0.94)	0.89 (0.86–0.92)	0.87 (0.84–0.90)	0.89 (0.85–0.92)	<0.001	0.85 (0.82–0.88)
Fully adjusted model^c^	Ref	0.94 (0.91–0.98)	0.95 (0.91–0.99)	0.94 (0.90–0.98)	0.96 (0.92–1.01)	<0.001	0.92 (0.88–0.97)
**Free testosterone**							
No. of events (%)	6,761 (23.2)	5,879 (20.2)	5,279 (18.1)	4,776 (16.4)	4,053 (13.9)		
Basic model^b^	Ref	0.95 (0.91–0.98)	0.93 (0.90–0.96)	0.94 (0.90–0.97)	0.94 (0.90–0.98)	<0.001	0.90 (0.86–0.94)
Fully adjusted model^c^	Ref	0.98 (0.94–1.01)	0.97 (0.94–1.01)	0.99 (0.95–1.02)	0.99 (0.95–1.03)	0.150	0.97 (0.93–1.01)
**Women (*n* = 147,733)**							
**Total testosterone**							
No. of events (%)	3,936 (13.3)	3,869 (13.1)	3,742 (12.7)	3,608 (12.2)	3,808 (12.9)		
Basic model^b^	Ref	1.03 (0.99–1.08)	1.04 (0.99–1.08)	1.04 (0.99–1.08)	1.14 (1.09–1.19)	<0.001	1.09 (1.06–1.12)
Fully adjusted model^c^	Ref	1.02 (0.98–1.07)	1.02 (0.97–1.07)	1.01 (0.96–1.05)	1.08 (1.04–1.13)	0.001	1.05 (1.02–1.08)
**Free testosterone**							
No. of events (%)	3,621 (12.3)	3,633 (12.3)	3,675 (12.4)	3,838 (13.0)	4,196 (14.2)		
Basic model^b^	Ref	1.01 (0.97–1.06)	1.05 (1.00–1.10)	1.12 (1.07–1.18)	1.27 (1.22–1.33)	<0.001	1.16 (1.13–1.19)
Fully adjusted model^c^	Ref	0.99 (0.95–1.04)	1.01 (0.96–1.06)	1.04 (1.00–1.09)	1.12 (1.07–1.18)	<0.001	1.08 (1.05–1.11)

Quintile cutoff points in men were 9.13, 10.95, 12.69, and 14.96 nmol/L for TT and 167.46, 195.55, 222.69, and 258.61 pmol/L for FT; quintile cutoff points in women were 0.67, 0.90, 1.15, and 1.48 nmol/L for TT and 7.50, 10.63, 14.30, and 19.94 pmol/L for FT.

CI, confidence interval; n/No., number; Ref, reference; SD, standard deviation; IPAQ, International Physical Activity Questionnaire; CCVD, cardiac-cerebral vascular disease; HRT, hormone replacement therapy; SHBG, sex hormone-binding globulin.

a The HRs of each group were compared with those in the bottom quintiles.

b Basic model: adjusted for age and menopause (for women).

c Fully adjusted model: further adjusted for college or university degree, Townsend deprivation index, body mass index, smoking status, alcohol drinking, IPAQ group, healthy diet, family history of diseases (CCVD or cancer), use of aspirin/ibuprofen, and HRT (for women). SHBG was additionally adjusted in total testosterone.

**Figure 1:**
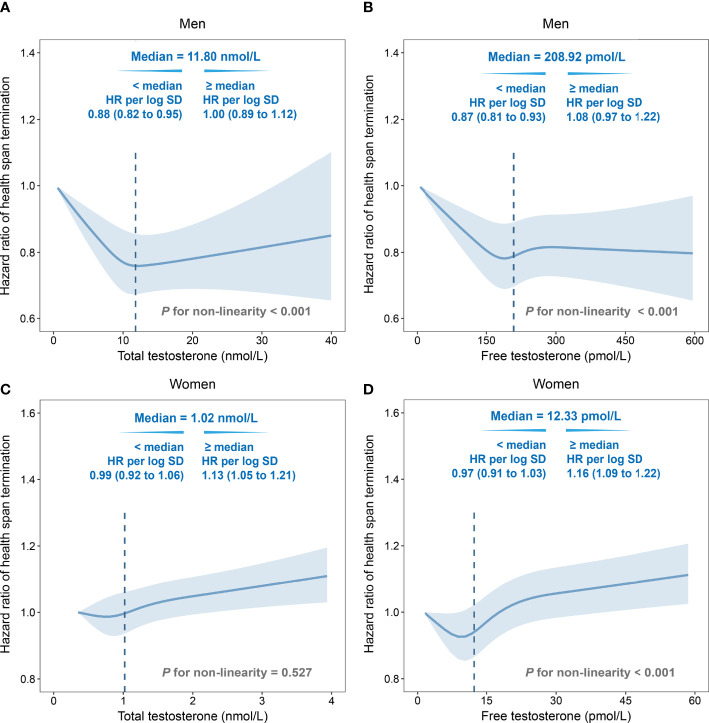
Non-linear associations of total testosterone/free testosterone (TT/FT) with risk of health span termination by multivariate cubic regression splines. **(A)** TT in men, **(B)** FT in men, **(C)** TT in women, and **(D)** FT in women. Solid lines were point estimation of hazard ratio, and shaded regions represented the 95% CIs. The dotted lines were the median values. Fully adjusted model: age, menopause status (for women), college or university degree, deprivation index, body mass index, smoking status, alcohol drinking, IPAQ group, healthy diet, family history of cardiac-cerebral vascular disease (CCVD) or cancer, use of aspirin/ibuprofen, and hormone replacement therapy (for women). SHBG was additionally adjusted in total testosterone. HR, hazard ratio; SD, standard deviation.

Non-linear relationships of testosterone and the risk of HST were observed for both genders. In men, an approximate L-shape curvilinear relationship was observed, both in TT and FT, using multivariate cubic models (**Figures 1A, B**). After natural log transformation for TT and FT concentrations, negative associations were observed below the corresponding medians (TT, HR: 0.88, 95% CI: 0.82–0.95; FT, HR: 0.87, 95% CI: 0.81–0.93). Interestingly, TT and FT had an inverse association with HST risk in women. The relationship was linear for TT, while it was more like a J-shape for FT (**Figures 1C, D**). Above the medians, HST risks were positively correlated with TT (HR: 1.13, 95% CI: 1.05–1.21) and FT (HR: 1.16, 95% CI: 1.09–1.22).

Due to the complexity of health span, we further explored the relationship between testosterone and cause-specific incidence. In men, testosterone was observed to have decreased the death, COPD, dementia, diabetes, and lung cancer risk, as well as the overall risk of non-cancer events, but increased the prostate cancer and myocardial infarction risk (**Supplementary Table 5**). In women, testosterone was positively associated with death, breast cancer, ovary cancer, endometrial cancer, and overall cancer, as well as dementia (total testosterone only) and diabetes (free testosterone only), but negatively related to COPD (**Supplementary Table 6**). In the stratified analyses, the associations of HST with testosterone were largely consistent across subgroups, except alcohol intake and Townsend deprivation index in men (**Supplementary Table 7**) and women with a family history of CCVD (**Supplementary Table 8**). In the joint analyses between TT and SHBG, the HST risk increased from 10% to 25% as the increase of TT level in women with the lowest strata of SHBG (**Supplementary Figure 3**).

**Figure 2:**
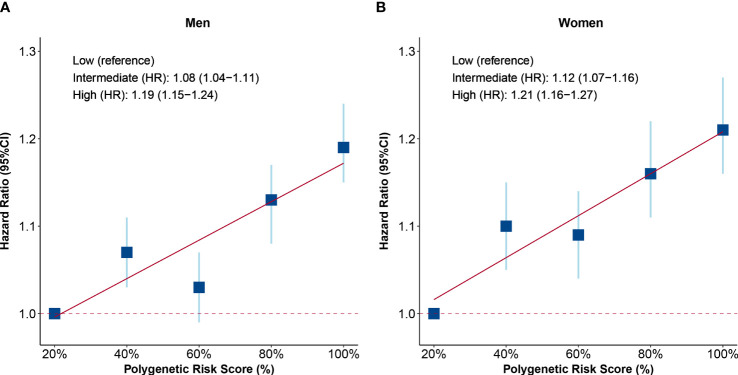
Associations of polygenetic risk score (PRS) with risk of health span termination. **(A)** PRS in men and **(B)** PRS in women. Participants were divided into five groups according to the quintiles of PRS, and the HRs of each quintile were compared with those in the bottom quintiles. The level of PRS was further divided into three groups: low (quintile 1), intermediate (quintiles 2–4), and high (quintile 5). The blue squares are point estimation of HRs and error bars are 95% CIs. Fully adjusted model: age, menopause status (for women), college or university degree, deprivation index, body mass index, smoking status, alcohol drinking, IPAQ group, healthy diet, family history of CCVD or cancer, use of aspirin/ibuprofen, use of hormone replacement therapy (for women), the top 10 principal components of ancestry, and genotyping chip. HR, hazard ratio; CI, confidential interval.

To explore the associations between genetic factors and health span, a polygenetic risk score was constructed by 12 SNPs of health span to evaluate individual genetic risk. As shown by the distribution of polygenetic risk scores, both men and women with HST showed a higher genetic risk (**Supplementary Figure 4**). Furthermore, we found a positive relationship between PRS and HST risk. Participants from both genders with high genetic risk tended to terminate health span prematurely, when compared with those with low genetic risk (men: HR: 1.19, 95% CI: 1.15–1.24; women: HR: 1.21, 95% CI: 1.16–1.27, **Figure 2**). However, no interaction between PRS and testosterone was observed (*P* > 0.05), and the associations of testosterone with HST were unchanged after additionally adjusting for PRS (**Supplementary Tables 9, 10**).

**Figure 3:**
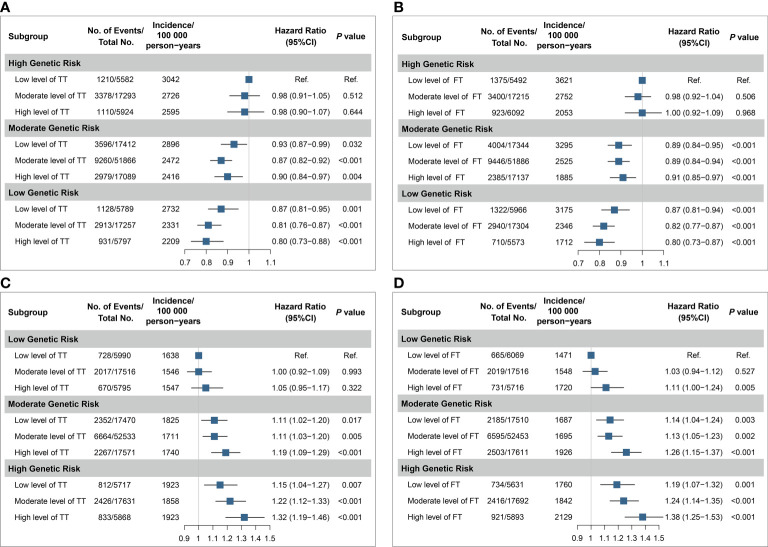
Joint analyses of testosterone and genetic categories for risk of health span termination. The joint effects for health span termination were estimated according to TT of men **(A)**, FT of men **(B)**, TT of women **(C)**, FT of women **(D)**, and PRS categories, respectively. The blue squares are point estimation of HRs and error bars are 95% CIs. The level of testosterone was divided into three groups: low (quintile 1), moderate (quintiles 2–4), and high (quintile 5), and the categories of PRS were similar. Each group was compared with the participants with low level of testosterone and high genetic risk in men, while the combination of low level of testosterone and low genetic risk was the reference in women. Fully adjusted model: age, menopause status (for women), college or university degree, deprivation index, body mass index, smoking status, alcohol drinking, IPAQ group, healthy diet, family history of CCVD or cancer, use of aspirin/ibuprofen, use of hormone replacement therapy (for women), the top 10 principal components of ancestry, and genotyping chip. SHBG was additionally adjusted in total testosterone. HR, hazard ratio; CI, confidential interval; Ref., reference; No., number; TT, total testosterone; FT, free testosterone.

Then, we found a joint effect of genetic risk and TT/FT. Specifically, compared with men with high genetic risk and low level of TT/FT, a 20% lower risk was observed in those with both low genetic risk and high level of TT/FT (TT, HR: 0.80, 95% CI: 0.73–0.88; FT, HR: 0.80, 95% CI: 0.73–0.87, **Figures 3A, B**). Besides, the HST risk increased with both genetic risk and testosterone concentration in women. Women with high genetic risk and testosterone level showed the highest risk in subgroups (TT, HR: 1.32, 95% CI: 1.19–1.46; FT, HR: 1.38, 95% CI: 1.25–1.53, **Figures 3C, D**).

Finally, sensitivity analyses showed consistent results after excluding participants with HST during the first 2 years of follow-up (**Supplementary Table 11**). When we excluded the participants with poor self-reported health status at baseline, the associations were also consistent (**Supplementary Table 12**). Moreover, after excluding participants with outliers of testosterone level (top 1% and bottom 1%), we still observed similar associations (**Supplementary Table 13**). After additional adjustment for fasting time or menstrual cycle proxy factors, similar associations were also presented (**Supplementary Tables 14, 15**).

## Discussion

In this large-scale cohort study, comprised of 145,481 men and 147,733 women from the UK Biobank, we observed sex-specific associations between serum testosterone and HST risk. The findings suggested that total testosterone might decrease the risk of health span termination in men. Inversely, women with a high level of TT or FT were at a higher risk of HST. Furthermore, genetic factors increased the risk of premature end of health span to some extent. Although no interplay between testosterone and genetic factors was observed, participants with both high genetic risk and abnormal levels of testosterone (low level in men and high level in women) had the highest HST risk.

Despite the scarcity of direct evidence in the recent two decades relating testosterone and health span, a growing number of epidemiological studies indicated that high levels of testosterone might be protective for all-cause mortality in men (12, 25), but inversely in women (13), which is consistent with our cause-specific findings. Similar to a prospective study from Denmark (10), we observed that the risk of overall cancer for men was not modified by testosterone levels. However, some researchers indicated that high testosterone might be a risk factor for prostate cancer (11). For women, the effects of endogenous testosterone might increase the risk of common gynecological cancer, such as breast (26) and endometrial cancer (27), supporting our cause-specific results. Metabolic syndrome was considered as a critical risk factor for common cancers (28). A high level of testosterone had been proven beneficial for metabolic syndrome risk in men but harmful in women (9, 29, 30), which supported our findings. Additionally, recent researches pointed out that testosterone deficiency in men was associated with increased risk of non-cancer chronic disease, such as cardiovascular disease (8, 31), type 2 diabetes (29, 32) and dementia (33, 34), as well as exacerbated the condition of COPD patients (35). In women, early studies revealed an inverse association of testosterone with increased CVD risk (36, 37), while it was not observed in the recent Rotterdam study (38). Although testosterone may be safe for myocardial function at physiological levels, a toxic effect also exists at supraphysiologic dose (39). As mentioned above, higher levels of testosterone are unfavorable to the metabolism of women and increase their risk of type 2 diabetes (30, 40). Thus, these pieces of evidence support that a sex-specific association indeed exists between testosterone and HST risk.

Except for endocrine factors, genetic variations also play an important role in the determination of individual health span (14). PRS is a simple approach for summing risks across multiple susceptibility loci, aiming at classifying populations with distinct risk levels to drive clinical or personal decision-making (15). Although similar longevity genome-wide association studies (41, 42) exist for a long time, studies that have assessed the predictive performance of PRS on longevity or health span remain scarce. Besides, the small sample size of a former study could affect the strength of the findings (43). In our study, PRS based on 12 SNPs from nearly 300,000 participants stratifies the participants into distinct HST risk strata clearly, indicating GWAS-derived PRS is a promising tool. The effectiveness of PRS still requires independent validation in other populations, as conducted in a large-scale prospective cohort study that applied the PRS to the CKB cohort for re-evaluation (16).

By summarizing the existing evidence related to death, cancer, and other non-cancer events, we indirectly discuss the sex-specific roles of testosterone in maintaining the period of health span. In view of a potential hazard of high concentration in prostate cancer (11) or other diseases in men, the negative association of testosterone with HST we observed in this study might be offset to some degree, accounting for non-significance in quintile 5 of TT. Thus, we conservatively suggest that this association is more stable at an intermediate dose (the second to fourth quintiles, Q2–Q4) in men, which is consistent with the guidelines by the European Endocrine Society that recommended achieving testosterone concentration in the mid-normal range during treatment (44). Previous studies indicated that a loss of testosterone due to aging contributed to the onset of physical frailty, such as triggering muscle loss, muscle weakness, and decreased functional performance, which eventually limited the lifespan in both genders (7, 45). Hence, an appropriate dose of testosterone is not necessarily harmful to women; our results also showed that HST risk only obviously increased at a high concentration of TT or FT (the top quintile, Q5). Besides, individuals with a very high or very low level of testosterone are strongly associated with health status (46, 47). Some diseases, such as pituitary disorders (48), HIV (49), and hyperthyroidism (50), as well as obesity (51), may interfere the testosterone production, increasing the possibility of extreme level of occurrence. It is worth noting that these diseases have a possible influence on personal health span as well. Therefore, we respectively excluded the participants with self-reported poor health status or outliers in the sensitivity analyses to control the potential bias introduced by the extreme values.

Given that both endocrine and genetic factors may contribute to health span collectively, we assess the joint effects of genetic risk and testosterone. Despite the non-significant interaction effect of testosterone and genetic propensity on health span, we still find the combined associations greater than individually. This suggests that those participants both with abnormal testosterone levels (especially high level in women and low level in men) and high genetic risk are in a more dangerous situation and deserve more attention. Therefore, taking appropriate intervention measures early, such as testosterone replacement therapy (TRT) for men with testosterone deficiency or anti-testosterone therapy for women with a supraphysiologic dose of testosterone, is considerable to extend personal health span.

Over the past two decades, TRT has been widely used for low age-related levels of testosterone in men (52) or even for postmenopausal women with low sexual desire (53), so there is a chance for overuse in the population. Therefore, current mainstream guidelines are cautious about such therapy and are critical about the usage and concentration (54, 55). For a better understanding of the roles played by exogenous testosterone in health span and to better assist clinical or personal decision-making before testosterone utilization, it is a wise choice to set endogenous testosterone as an entry point of research. Hopefully soon, we will be able to screen populations suitable for testosterone therapy by utilizing PRS in risk prediction for precise prevention.

Several limitations deserve to be noticed. Firstly, a single measurement for testosterone at baseline may not reflect long-term exposure very well. However, some researchers consider it reliable in categorizing average levels, as it calculates the intraclass correlation coefficients (ICCs) between two measurements of TT that were 4 years apart in a subcohort of UKB (13). Secondly, although the Vermeulen formula is the most widely used among different methods, calculating FT is still possible to be over- or underestimated relative to laboratory measurement (56). Third, given the aromatization of free testosterone into estradiol (57), further adjustment for estradiol is necessary to evaluate the independent association of FT in women. However, it is infeasible to perform at the current phase because most postmenopausal women in the UK Biobank have estradiol levels below the threshold for detection and premenopausal women also have a high missing rate of estradiol. Finally, there is evidence of a “healthy volunteer” selection bias (58), which suggests that it is warranted to replicate our findings in other cohorts to determine the applicability of our findings to other populations.

In conclusion, this prospective cohort study revealed the sex-specific associations that testosterone was negatively associated with HST risk in men and positively associated with HST risk in women. Genetic factors increased the HST risk, which indicated that participants with abnormal testosterone levels (high level in women or low level in men) and high genetic risk should be the target for early prevention. Although our findings highlight the association between testosterone and health span, further mechanistic studies and prospective trials are warranted to explore the causation behind.

## Data Availability Statement

Publicly available datasets were analyzed in this study. Datasets are available from the UK Biobank *via* application to the UK Biobank.

## Ethics Statement

The studies involving human participants were reviewed and approved by the North West Multi-Centre Research Ethics Committee (http://www.ukbiobank.ac.uk/ethics/). The patients/participants provided their written informed consent to participate in this study.

## Author Contributions

JD supervised the entire project and designed the work. XZ conducted the statistical analysis and wrote the first draft. SL, NW, and TH helped apply for permission to use data and contributed to data management. MS and JF offered statistical support during the study. MZ, CW, DH, and YJ critically revised the manuscript for important intellectual content. All authors contributed to the article and approved the submitted version.

## Funding

This work was supported by the National Natural Science Foundation of China (Grant ID: 81941020) as part of a project entitled “Identification of longevity-related genetic variations and their biological mechanisms based on a natural population cohort”.

## Conflict of Interest

The authors declare that the research was conducted in the absence of any commercial or financial relationships that could be construed as a potential conflict of interest.

## Publisher’s Note

All claims expressed in this article are solely those of the authors and do not necessarily represent those of their affiliated organizations, or those of the publisher, the editors and the reviewers. Any product that may be evaluated in this article, or claim that may be made by its manufacturer, is not guaranteed or endorsed by the publisher.
