# Supplementary material for: Sex-Specific Associations of Testosterone and Genetic Factors With Health Span
Source: Front Endocrinol (Lausanne). 2021 Nov 25;12:773464. doi: 10.3389/fendo.2021.773464 (PMC8655098; doi:10.3389/fendo.2021.773464)
Supplement: Supplementary file 1 [file DataSheet_1.docx]

**Supplementary Materials**

**Content**

| Supplementary Methods | Ascertainment of covariates |
| --- | --- |
| Supplementary Table 1 | Disease codes (ICD 10) for health span composition |
| Supplementary Table 2 | SNP list of health span identified in the study of *Zenin* et al. (2019) |
| Supplementary Table 3 | Number and composition ratio of sub-events for construction of health span |
| Supplementary Table 4 | Baseline characteristics of participants according to quintile of total testosterone |
| Supplementary Table 5 | Associations of total testosterone and free testosterone with cause-specific incidence in men (n=145,481) |
| Supplementary Table 6 | Associations of total testosterone and free testosterone with cause-specific incidence in women (n=147,733) |
| Supplementary Table 7 | Stratified analysis of associations between total testosterone, free testosterone and risk of health span termination in men |
| Supplementary Table 8 | Stratified analysis of associations between total testosterone, free testosterone and risk of health span termination in women |
| Supplementary Table 9 | Associations of total or free testosterone with incident health span termination with adjustment for PRS in men |
| Supplementary Table 10 | Associations of total or free testosterone with incident health span termination with adjustment for PRS in women |
| Supplementary Table 11 | Sensitivity analyses excluding participants with health span end within the first two years of follow-up (*n*=5,749 in men, *n*=4,100 in women) for the associations between total testosterone, free testosterone and HST risk |
| Supplementary Table 12 | Sensitivity analyses excluding participants with self-reported health status at baseline (*n*=4,164 in men, *n*=3,264 in women) for the associations between testosterone, free testosterone and HST risk |
| Supplementary Table 13 | Sensitivity analyses excluding participants with outliers for the associations between testosterone, free testosterone and HST risk |
| Supplementary Table 14 | Sensitivity analyses adjusting fasting time for the associations between total testosterone, free testosterone and HST risk |
| Supplementary Table 15 | Sensitivity analyses adjusting menstrual cycle proxy factors for the associations between testosterone, free testosterone and HST risk in women (*n*=147,733) |
| Supplementary Figure 1 | Study design and workflow |
| Supplementary Figure 2 | Distribution of total testosterone and free testosterone in men and women |
| Supplementary Figure 3 | Joint analyses of total testosterone and sex hormone binding globulin with health span in the fully-adjusted models |
| Supplementary Figure 4 | Distribution of polygenetic risk score between terminated and unterminated health span participants |

**Supplementary Methods. Ascertainment of covariates**

Socio-demographic, behavioral risk factors and other confounding factors, which could potentially confuse the association between serum testosterone and health span were considered as covariates. The basic model was adjusted for age at blood draw (continuous), menopause status (yes and no). Fully-adjusted model was further adjusted for Townsend deprivation index (continuous), education (have college or university degree and other), body mass index (BMI, continuous), smoking status (never, previous and current), alcohol intake frequency (never, special occasions only, one to three times a month, once or twice a week, three or four times a week and daily or almost daily), International Physical Activity Questionnaire (IPAQ) activity group (high, moderate and low), healthy diet (yes and no), family history of cancer (yes and no), family history of cardiac-cerebral vascular disease (CCVD, yes and no), aspirin/ibuprofen use (yes, no) and hormone replacement therapy use (HRT, yes and no). To clarify the independent effect of TT, sex hormone binding globulin (SHBG, continuous) was included as the additional adjustment in the fully-adjusted model of TT. Moreover, the top 10 genetic principal components (PC1-10, continuous) and genotyping chip (Affymetrix UK BiLEVE Axiom and Affymetrix UK Biobank Axiom) were also adjusted in the genetic parts.

We coded missing data as a missing indicator category for categorical variables and used sex-specific medians to impute the missing value for continuous variables. All covariates had <2% missing, except physical activity (14.57% in men and 21.32% in women), family history of cancer (6.64% in men and 4.61% in women) and CCVD (5.38% in men and 3.54% in women), as well as menopause status (14.17%)

| **Supplementary Table 1. Disease codes (ICD 10) for health span composition** | | |
| --- | --- | --- |
| **Disease ^a^** | **ICD10 in-patient hospital  admissions data (UKB data category 2000)** | **Self-reported diagnoses obtained  via verbal interview (UKB data category 100074)** |
| CHF | I50 | 1076 |
| COPD | J44 | 1112 |
| MI | I21, I22, I23, I24, I25 | 1075 |
| Dementia | F00, F01, F02, F03, F04, F05 | 1263, 1258, 1259, 1260, 1261, 1262 |
| Diabetes | E10, E11, E12, E13, E14 | 1220, 1221, 1222, 1223, 1521 |
| Stroke | I60, I61, I62, I63, I64 | 1081, 1086, 1491, 1583 |

^a^: Cancer and death events were derived from National cancer registries (UKB data category 100092) and National death registries (UKB data category 100093). Abbreviations: ICD, international classification of diseases; CHF, congestive heart failure; COPD, chronic obstructive pulmonary disease; MI, myocardial infarction.

| **Supplementary Table 2. SNP list of health span identified in the study of *Zenin* et al. (2019)** | | | | | | | |
| --- | --- | --- | --- | --- | --- | --- | --- |
| **SNP** | **Chr** | **Position (bp)** | **EA** | **RA** | **EAF** | **beta** | ***P*** |
| rs10197246 | 2 | 202204741 | C | T | 0.734 | -0.033 | 3.67E-09 |
| rs12203592 | 6 | 396321 | T | C | 0.214 | 0.063 | 1.80E-25 |
| rs1049053 | 6 | 32634405 | T | C | 0.671 | 0.037 | 1.40E-11 |
| rs10455872 | 6 | 161010118 | G | A | 0.081 | 0.057 | 4.11E-10 |
| rs140570886 | 6 | 161013013 | C | T | 0.016 | 0.116 | 2.18E-08 |
| rs7859727 | 9 | 22102165 | T | C | 0.488 | 0.031 | 7.41E-10 |
| rs34872471 | 10 | 114754071 | C | T | 0.292 | 0.061 | 9.73E-29 |
| rs2860197 | 10 | 123351302 | A | G | 0.613 | -0.029 | 1.22E-08 |
| rs1126809 | 11 | 89017961 | A | G | 0.304 | 0.04 | 2.35E-13 |
| rs4784227 | 16 | 52599188 | T | C | 0.24 | 0.032 | 3.02E-08 |
| rs4268748 | 16 | 90026512 | C | T | 0.311 | 0.038 | 1.55E-12 |
| rs159428 | 20 | 31099311 | C | T | 0.527 | 0.028 | 2.36E-08 |

Abbreviations: SNP, single nucleotide polymorphism; Chr, chromsome; EA, effective allele; RA, reference allele; EAF, effect allele frequency; beta, regression coefficient estimate; *P*, *p*-value.

| **Supplementary Table 3. Number and composition ratio of sub-events for construction of health span** | | | | | | |
| --- | --- | --- | --- | --- | --- | --- |
| **Outcomes** | **Overall** | **%** | **Men** | **%** | **Women** | **%** |
| Cancer | 27,162 | 46.3 | 14,624 | 41.5 | 12,538 | 53.6 |
| MI | 10,448 | 17.8 | 7,472 | 21.2 | 2,976 | 12.7 |
| Death | 6,689 | 11.4 | 4,316 | 12.2 | 2,373 | 10.1 |
| Diabetes | 5,320 | 9.1 | 3,245 | 9.2 | 2,075 | 8.9 |
| COPD | 4,350 | 7.4 | 2,632 | 7.5 | 1,718 | 7.3 |
| Stroke | 2,666 | 4.5 | 1,675 | 4.8 | 991 | 4.2 |
| Dementia | 1,162 | 2.0 | 715 | 2.0 | 447 | 1.9 |
| CHF | 852 | 1.5 | 571 | 1.6 | 281 | 1.2 |
| Total ^a^ | 58,649 | 100.0 | 35,250 | 100.0 | 23,399 | 100.0 |

^a^: The total number only represents person-time of events, not the actual number of participants with health span termination. Abbreviations: ICD, international classification of diseases; CHF, congestive heart failure; COPD, chronic obstructive pulmonary disease; MI, myocardial infarction.

| **Supplementary Table 4. Baseline characteristics of participants according to quintile of total testosterone** | | | | | | |
| --- | --- | --- | --- | --- | --- | --- |
| **Characteristics** | **Total testosterone (nmol/L)** | | | | | |
|  | **Men (*n*=154,476)** | | | **Women (*n*=156,884)** | | |
|  | **Quintile 1  (0.36-9.13)** | **Quintile 3  (10.95-12.69)** | **Quintile 5  (14.97-53.14)** | **Quintile 1  (0.35-0.67)** | **Quintile 3 (0.90-1.15)** | **Quintile 5 (1.48-49.85)** |
| Events, No. (%) | 5,988 (20.58) | 5,255 (18.06) | 5,066 (17.40) | 3,936 (13.34) | 3,742 (12.66) | 3,808 (12.88) |
| Age, mean (SD), year | 56.42 (7.98) | 55.95 (8.15) | 55.36 (8.28) | 57.27 (7.51) | 55.58 (7.97) | 54.12 (8.31) |
| Follow-up time (SD), year | 7.13 (1.97) | 7.20 (1.89) | 7.22 (1.86) | 7.38 (1.70) | 7.41 (1.67) | 7.41 (1.68) |
| Townsend index, mean (SD) ^a^ | -1.56 (2.94) | -1.60 (2.94) | -1.23 (3.14) | -1.64 (2.86) | -1.57 (2.89) | -1.43 (2.96) |
| College or university degree, No. (%) | 9,555 (32.85) | 10,438 (35.88) | 10,335 (35.51) | 9,316 (31.57) | 9,707 (32.84) | 9,475 (32.05) |
| BMI, mean (SD), kg/m2 | 29.35 (4.51) | 27.47 (3.71) | 26.01 (3.46) | 26.37 (4.66) | 26.83 (4.93) | 27.78 (5.43) |
| Smoking status, No. (%) |  |  |  |  |  |  |
| Never | 14,163 (48.69) | 14,962 (51.43) | 15,097 (51.87) | 17,592 (59.62) | 17,752 (60.06) | 17,733 (59.99) |
| Previous | 12,003 (41.26) | 10,724 (36.86) | 9,178 (31.53) | 9,708 (32.90) | 9,184 (31.07) | 8,530 (28.86) |
| Current | 2,824 (9.71) | 3,314 (11.39) | 4,750 (16.32) | 2,099 (7.11) | 2,530 (8.56) | 3,219 (10.89) |
| Alcohol intake, No. (%) |  |  |  |  |  |  |
| Heavy | 15,466 (53.20) | 16,164 (55.61) | 15,350 (52.79) | 11,350 (38.49) | 11,719 (39.67) | 11,775 (39.85) |
| Moderate | 10,294 (35.41) | 10,037 (34.53) | 10,271 (35.33) | 11,868 (40.24) | 11,892 (40.26) | 11,814 (39.99) |
| Light | 3,311 (11.39) | 2,866 (9.86) | 3,454 (11.88) | 6,273 (21.27) | 5,927 (20.07) | 5,956 (20.16) |
| IPAQ group, No. (%) |  |  |  |  |  |  |
| High | 9,668 (33.23) | 11,049 (37.98) | 12,011 (41.26) | 9,371 (31.76) | 9,002 (30.46) | 8,458 (28.61) |
| Moderate | 9,623 (33.08) | 9,670 (33.24) | 9,049 (31.09) | 9,893 (33.53) | 10,037 (33.96) | 10,138 (34.30) |
| Low | 5,376 (18.48) | 4,326 (14.87) | 3,740 (12.85) | 3,852 (13.06) | 4,199 (14.21) | 4,555 (15.41) |
| Healthy diet, No. (%) | 18,789 (64.59) | 19,519 (67.10) | 19,260 (66.17) | 24,518 (83.10) | 24,233 (81.98) | 23,707 (80.20) |
| Family history of CCVD, No. (%) | 15,640 (53.76) | 15,262 (52.46) | 15,008 (51.56) | 17,499 (59.31) | 16,861 (57.04) | 16,749 (56.66) |
| Family history of cancer, No. (%) | 10,251 (35.24) | 10,132 (34.83) | 10,021 (34.43) | 10,566 (35.81) | 10,504 (35.54) | 10,270 (34.74) |
| Use of aspirin/ibuprofen, No. (%) | 7,323 (25.17) | 6,516 (22.40) | 5,871 (20.17) | 6,923 (23.46) | 7,101 (24.02) | 7,032 (23.79) |
| Ever used HRT, No. (%) | - | - | - | 12,943 (43.87) | 10,178 (34.43) | 8,069 (27.30) |
| Had menopause, No. (%) | - | - | - | 19,133 (64.85) | 17,436 (58.99) | 15,540 (52.57) |

The Kruskal-Wallis one-way ANOVA test for continuous variables and the Chi-squared test for categorical variables were used to calculate the *P* values across the quintile groups in both genders respectively; The variables listed all had a *P* value < 0.05, except 'use of aspirin/ibuprofen' in women. ^a^: Positive values of the index will indicate areas with high material deprivation, whereas those with negative values will indicate relative affluence. Abbreviations: BMI, Body mass index; IPAQ, international physical activity questionnaire; CCVD, cardiac-cerebral vascular disease; HRT, hormone replacement therapy.

| **Supplementary Table 5. Associations of total testosterone and free testosterone with cause-specific incidence in men (*n*=145,481)** | | | | | | | |
| --- | --- | --- | --- | --- | --- | --- | --- |
|  | **Hazard Ratio (95% CI)** | | | | | ***P* for trend** | **HR per log SD  increase** |
|  | **Quintile 1** | **Quintile 2** | **Quintile 3** | **Quintile 4** | **Quintile 5** |  |  |
| **Total testosterone** |  |  |  |  |  |  |  |
| Death events, N (%) | 934 (3.2) | 845 (2.9) | 768 (2.6) | 816 (2.8) | 953 (3.3) |  |  |
| Fully-adjusted model | ref | 0.90 (0.82-0.99) | 0.79 (0.72-0.88) | 0.80 (0.72-0.88) | 0.83 (0.74-0.93) | <0.001 | 0.79 (0.71-0.88) |
| Overall cancer, N (%) | 3,093 (10.6) | 2,930 (10.1) | 2,931 (10.1) | 2,886 (9.9) | 2,784 (9.6) |  |  |
| Fully-adjusted model | ref | 0.97 (0.92-1.02) | 0.98 (0.93-1.04) | 0.99 (0.93-1.04) | 0.99 (0.93-1.05) | 0.913 | 1.00 (0.93-1.06) |
| Colorectal cancer, N (%) | 267 (0.9) | 275 (0.9) | 248 (0.9) | 217 (0.7) | 206 (0.7) |  |  |
| Fully-adjusted model | ref | 1.10 (0.92-1.30) | 1.03 (0.86-1.23) | 0.93 (0.77-1.14) | 0.95 (0.76-1.18) | 0.331 | 0.90 (0.72-1.12) |
| Lung cancer, N (%) | 144 (0.5) | 120 (0.4) | 123 (0.4) | 117 (0.4) | 125 (0.4) |  |  |
| Fully-adjusted model | ref | 0.83 (0.65-1.06) | 0.83 (0.64-1.06) | 0.73 (0.56-0.95) | 0.70 (0.52-0.93) | 0.001 | 0.64 (0.49-0.84) |
| Prostatic cancer, N (%) | 832 (2.9) | 780 (2.7) | 784 (2.7) | 816 (2.8) | 756 (2.6) |  |  |
| Fully-adjusted model | ref | 0.99 (0.90-1.09) | 1.03 (0.93-1.14) | 1.13 (1.02-1.26) | 1.16 (1.03-1.31) | <0.001 | 1.25 (1.10-1.42) |
| Non-cancer events, N (%) | 3,319 (11.4) | 2,681 (9.2) | 2,562 (8.8) | 2,444 (8.4) | 2,404 (8.3) |  |  |
| Fully-adjusted model | ref | 0.90 (0.85-0.95) | 0.90 (0.86-0.96) | 0.90 (0.85-0.95) | 0.92 (0.86-0.98) | <0.001 | 0.86 (0.80-0.91) |
| CHF, N (%) | 154 (0.5) | 107 (0.4) | 84 (0.3) | 115 (0.4) | 112 (0.4) |  |  |
| Fully-adjusted model | ref | 0.81 (0.63-1.04) | 0.68 (0.52-0.90) | 0.97 (0.74-1.27) | 0.96 (0.71-1.30) | 0.351 | 0.87 (0.64-1.17) |
| COPD, N (%) | 601 (2.1) | 495 (1.7) | 437 (1.5) | 501 (1.7) | 599 (2.1) |  |  |
| Fully-adjusted model | ref | 0.83 (0.74-0.94) | 0.71 (0.63-0.81) | 0.75 (0.66-0.86) | 0.75 (0.65-0.86) | <0.001 | 0.67 (0.59-0.77) |
| MI, N (%) | 1,635 (5.6) | 1,525 (5.2) | 1,534 (5.3) | 1,442 (5.0) | 1,337 (4.6) |  |  |
| Fully-adjusted model | ref | 1.04 (0.97-1.12) | 1.11 (1.03-1.20) | 1.11 (1.02-1.20) | 1.11 (1.01-1.21) | 0.012 | 1.12 (1.03-1.23) |
| Dementia, N (%) | 170 (0.6) | 140 (0.5) | 115 (0.4) | 140 (0.5) | 151 (0.5) |  |  |
| Fully-adjusted model | ref | 0.81 (0.64-1.01) | 0.64 (0.50-0.82) | 0.74 (0.58-0.94) | 0.70 (0.53-0.91) | 0.006 | 0.70 (0.54-0.90) |
| Diabetes, N (%) | 1,217 (4.2) | 693 (2.4) | 552 (1.9) | 446 (1.5) | 338 (1.2) |  |  |
| Fully-adjusted model | ref | 0.82 (0.74-0.90) | 0.79 (0.71-0.88) | 0.76 (0.67-0.85) | 0.72 (0.63-0.84) | <0.001 | 0.73 (0.65-0.82) |
| Stroke, N (%) | 331 (1.1) | 321 (1.1) | 323 (1.1) | 339 (1.2) | 362 (1.2) |  |  |
| Fully-adjusted model | ref | 1.01 (0.87-1.18) | 1.02 (0.87-1.20) | 1.07 (0.91-1.26) | 1.12 (0.93-1.34) | 0.472 | 1.07 (0.89-1.29) |
| **Free testosterone** |  |  |  |  |  |  |  |
| Death events, N (%) | 1,225 (4.2) | 939 (3.2) | 802 (2.8) | 744 (2.6) | 606 (2.1) |  |  |
| Fully-adjusted model | ref | 0.89 (0.81-0.97) | 0.85 (0.78-0.93) | 0.89 (0.81-0.98) | 0.87 (0.79-0.97) | <0.001 | 0.79 (0.71-0.88) |
| Overall cancer, N (%) | 3,579 (12.3) | 3,264 (11.2) | 3,005 (10.3) | 2,599 (8.9) | 2,177 (7.5) |  |  |
| Fully-adjusted model | ref | 1.02 (0.97-1.07) | 1.04 (0.99-1.10) | 1.02 (0.97-1.07) | 1.04 (0.99-1.10) | 0.349 | 1.03 (0.97-1.09) |
| Colorectal cancer, N (%) | 314 (1.1) | 265 (0.9) | 233 (0.8) | 229 (0.8) | 172 (0.6) |  |  |
| Fully-adjusted model | ref | 0.96 (0.81-1.13) | 0.94 (0.80-1.12) | 1.05 (0.88-1.25) | 0.96 (0.80-1.17) | 0.879 | 0.98 (0.80-1.21) |
| Lung cancer, N (%) | 192 (0.7) | 154 (0.5) | 106 (0.4) | 95 (0.3) | 82 (0.3) |  |  |
| Fully-adjusted model | ref | 0.98 (0.79-1.21) | 0.78 (0.61-0.99) | 0.81 (0.63-1.04) | 0.86 (0.66-1.12) | 0.001 | 0.66 (0.51-0.85) |
| Prostatic cancer, N (%) | 940 (3.2) | 928 (3.2) | 858 (2.9) | 677 (2.3) | 565 (1.9) |  |  |
| Fully-adjusted model | ref | 1.12 (1.03-1.23) | 1.19 (1.08-1.30) | 1.09 (0.99-1.21) | 1.17 (1.05-1.30) | <0.001 | 1.27 (1.13-1.43) |
| Non-cancer events, N (%) | 3,619 (12.4) | 2,956 (10.2) | 2,514 (8.6) | 2,346 (8.1) | 1,975 (6.8) |  |  |
| Fully-adjusted model | ref | 0.95 (0.91-1.00) | 0.91 (0.86-0.95) | 0.95 (0.90-1.01) | 0.95 (0.90-1.01) | 0.005 | 0.92 (0.86-0.97) |
| CHF, N (%) | 178 (0.6) | 125 (0.4) | 105 (0.4) | 94 (0.3) | 70 (0.2) |  |  |
| Fully-adjusted model | ref | 0.91 (0.72-1.14) | 0.92 (0.72-1.17) | 0.99 (0.76-1.28) | 0.97 (0.73-1.29) | 0.379 | 0.88 (0.67-1.17) |
| COPD, N (%) | 835 (2.9) | 541 (1.9) | 500 (1.7) | 405 (1.4) | 352 (1.2) |  |  |
| Fully-adjusted model | ref | 0.77 (0.69-0.86) | 0.83 (0.74-0.92) | 0.76 (0.67-0.86) | 0.78 (0.68-0.89) | <0.001 | 0.66 (0.59-0.75) |
| MI, N (%) | 1,812 (6.2) | 1,684 (5.8) | 1,419 (4.9) | 1,367 (4.7) | 1,191 (4.1) |  |  |
| Fully-adjusted model | ref | 1.07 (1.00-1.14) | 1.01 (0.94-1.08) | 1.09 (1.02-1.17) | 1.14 (1.06-1.23) | 0.002 | 1.14 (1.05-1.24) |
| Dementia, N (%) | 252 (0.9) | 152 (0.5) | 117 (0.4) | 116 (0.4) | 79 (0.3) |  |  |
| Fully-adjusted model | ref | 0.74 (0.61-0.91) | 0.68 (0.54-0.84) | 0.82 (0.65-1.02) | 0.75 (0.58-0.97) | 0.002 | 0.69 (0.54-0.87) |
| Diabetes, N (%) | 1,001 (3.4) | 712 (2.4) | 584 (2.0) | 540 (1.9) | 409 (1.4) |  |  |
| Fully-adjusted model | ref | 0.90 (0.82-1.00) | 0.85 (0.77-0.94) | 0.88 (0.79-0.98) | 0.76 (0.67-0.86) | 0.007 | 0.86 (0.76-0.96) |
| Stroke, N (%) | 410 (1.4) | 400 (1.4) | 312 (1.1) | 309 (1.1) | 245 (0.8) |  |  |
| Fully-adjusted model | ref | 1.12 (0.97-1.28) | 0.97 (0.84-1.13) | 1.09 (0.94-1.26) | 1.04 (0.88-1.22) | 0.580 | 1.05 (0.88-1.25) |

Quintile cutoff points were 9.13, 10.95, 12.69, 14.96 nmol/L for TT and 167.46, 195.55, 222.69, 258.61 pmol/L for FT in men. The HRs of each group were compared with those in the bottom quintiles. Non-cancer events included CHF, MI, COPD, stroke, dementia and diabetes. Fully-adjusted model was as mentioned in Methods. ICD10 of cancer: colorectal cancer, C18-20; lung cancer, C33-34; prostatic cancer, C61. Abbreviations: HR, hazard ratio; CI, confidence interval; N, number; ref, reference; SD, standard deviation; ref, reference; CHF, congestive heart failure; COPD, chronic obstructive pulmonary disease; MI, myocardial infarction.

| **Supplementary Table 6. Associations of total testosterone and free testosterone with cause-specific incidence in women (*n*=147,733)** | | | | | | | |
| --- | --- | --- | --- | --- | --- | --- | --- |
|  | **Hazard Ratio (95% CI)** | | | | | ***P* for trend** | **HR per log SD increase** |
|  | **Quintile 1** | **Quintile 2** | **Quintile 3** | **Quintile 4** | **Quintile 5** |  |  |
| **Total testosterone** |  |  |  |  |  |  |  |
| Death events, N (%) | 481 (1.6) | 498 (1.7) | 434 (1.5) | 459 (1.5) | 501 (1.7) |  |  |
| Fully-adjusted model | ref | 1.09 (0.96-1.24) | 0.99 (0.87-1.12) | 1.08 (0.95-1.23) | 1.21 (1.07-1.37) | 0.003 | 1.14 (1.04-1.24) |
| Overall cancer, N (%) | 2,504 (8.5) | 2,581 (8.8) | 2,487 (8.4) | 2,415 (8.2) | 2,551 (8.6) |  |  |
| Fully-adjusted model | ref | 1.07 (1.01-1.13) | 1.06 (1.01-1.12) | 1.06 (1.01-1.13) | 1.16 (1.10-1.23) | <0.001 | 1.10 (1.06-1.14) |
| Colorectal cancer, N (%) | 176 (0.6) | 155 (0.5) | 162 (0.5) | 127 (0.4) | 154 (0.5) |  |  |
| Fully-adjusted model | ref | 0.93 (0.75-1.15) | 1.01 (0.81-1.25) | 0.83 (0.66-1.04) | 1.04 (0.84-1.30) | 0.997 | 1.00 (0.86-1.16) |
| Lung cancer, N (%) | 111 (0.4) | 111 (0.4) | 88 (0.3) | 89 (0.3) | 99 (0.3) |  |  |
| Fully-adjusted model | ref | 1.08 (0.83-1.40) | 0.88 (0.66-1.17) | 0.92 (0.70-1.22) | 1.05 (0.79-1.38) | 0.593 | 1.05 (0.87-1.27) |
| Breast cancer, N (%) | 635 (2.2) | 680 (2.3) | 674 (2.3) | 673 (2.3) | 799 (2.7) |  |  |
| Fully-adjusted model | ref | 1.09 (0.98-1.22) | 1.09 (0.98-1.22) | 1.11 (0.99-1.23) | 1.33 (1.19-1.48) | <0.001 | 1.20 (1.12-1.29) |
| Endometrial cancer, N (%) | 61 (0.2) | 101 (0.3) | 107 (0.4) | 114 (0.4) | 142 (0.5) |  |  |
| Fully-adjusted model | ref | 1.66 (1.21-2.29) | 1.76 (1.28-2.41) | 1.88 (1.38-2.57) | 2.26 (1.67-3.07) | <0.001 | 1.54 (1.29-1.84) |
| Ovarian cancer, N (%) | 72 (0.2) | 68 (0.2) | 63 (0.2) | 66 (0.2) | 78 (0.3) |  |  |
| Fully-adjusted model | ref | 0.98 (0.70-1.37) | 0.94 (0.67-1.32) | 1.03 (0.73-1.44) | 1.26 (0.91-1.74) | 0.014 | 1.32 (1.06-1.66) |
| Non-cancer events, N (%) | 1,562 (5.3) | 1,417 (4.8) | 1,398 (4.7) | 1,326 (4.5) | 1,413 (4.8) |  |  |
| Fully-adjusted model | ref | 0.95 (0.88-1.02) | 0.97 (0.90-1.04) | 0.93 (0.87-1.01) | 1.00 (0.93-1.07) | 0.919 | 1.00 (0.95-1.05) |
| CHF, N (%) | 54 (0.2) | 75 (0.3) | 46 (0.2) | 44 (0.1) | 62 (0.2) |  |  |
| Fully-adjusted model | ref | 1.41 (0.99-2.01) | 0.87 (0.59-1.30) | 0.84 (0.56-1.25) | 1.13 (0.78-1.64) | 0.638 | 0.94 (0.73-1.21) |
| COPD, N (%) | 424 (1.4) | 337 (1.1) | 363 (1.2) | 289 (1.0) | 305 (1.0) |  |  |
| Fully-adjusted model | ref | 0.83 (0.72-0.96) | 0.94 (0.81-1.08) | 0.75 (0.65-0.88) | 0.80 (0.69-0.93) | 0.003 | 0.85 (0.77-0.95) |
| MI, N (%) | 694 (2.4) | 585 (2.0) | 596 (2.0) | 558 (1.9) | 543 (1.8) |  |  |
| Fully-adjusted model | ref | 0.89 (0.80-0.99) | 0.96 (0.86-1.07) | 0.94 (0.84-1.05) | 0.94 (0.84-1.05) | 0.236 | 0.95 (0.88-1.03) |
| Dementia, N (%) | 94 (0.3) | 82 (0.3) | 91 (0.3) | 81 (0.3) | 99 (0.3) |  |  |
| Fully-adjusted model | ref | 0.94 (0.70-1.27) | 1.12 (0.84-1.50) | 1.08 (0.80-1.45) | 1.39 (1.04-1.84) | 0.012 | 1.28 (1.06-1.56) |
| Diabetes, N (%) | 391 (1.3) | 420 (1.4) | 386 (1.3) | 377 (1.3) | 501 (1.7) |  |  |
| Fully-adjusted model | ref | 1.10 (0.96-1.26) | 1.01 (0.87-1.16) | 0.95 (0.82-1.10) | 1.18 (1.03-1.35) | 0.052 | 1.10 (1.00-1.20) |
| Stroke, N (%) | 209 (0.7) | 201 (0.7) | 183 (0.6) | 191 (0.6) | 207 (0.7) |  |  |
| Fully-adjusted model | ref | 1.02 (0.84-1.24) | 0.97 (0.80-1.19) | 1.06 (0.87-1.29) | 1.19 (0.98-1.44) | 0.119 | 1.11 (0.97-1.27) |
| **Free testosterone** |  |  |  |  |  |  |  |
| Death events, N (%) | 473 (1.6) | 472 (1.6) | 461 (1.6) | 462 (1.6) | 505 (1.7) |  |  |
| Fully-adjusted model | ref | 1.00 (0.88-1.14) | 0.99 (0.87-1.12) | 0.99 (0.87-1.13) | 1.08 (0.95-1.23) | 0.462 | 1.03 (0.95-1.11) |
| Overall cancer, N (%) | 2,464 (8.3) | 2,448 (8.3) | 2,473 (8.4) | 2,559 (8.7) | 2,594 (8.8) |  |  |
| Fully-adjusted model | ref | 1.00 (0.95-1.06) | 1.03 (0.98-1.09) | 1.08 (1.02-1.15) | 1.12 (1.06-1.19) | <0.001 | 1.08 (1.05-1.12) |
| Colorectal cancer, N (%) | 176 (0.6) | 154 (0.5) | 134 (0.5) | 158 (0.5) | 152 (0.5) |  |  |
| Fully-adjusted model | ref | 0.89 (0.72-1.11) | 0.80 (0.63-1.00) | 0.96 (0.77-1.19) | 0.95 (0.75-1.19) | 0.818 | 0.98 (0.86-1.12) |
| Lung cancer, N (%) | 103 (0.3) | 104 (0.4) | 80 (0.3) | 105 (0.4) | 106 (0.4) |  |  |
| Fully-adjusted model | ref | 1.04 (0.79-1.37) | 0.82 (0.61-1.11) | 1.09 (0.83-1.44) | 1.12 (0.84-1.49) | 0.451 | 1.06 (0.91-1.25) |
| Breast cancer, N (%) | 578 (2.0) | 613 (2.1) | 733 (2.5) | 736 (2.5) | 801 (2.7) |  |  |
| Fully-adjusted model | ref | 1.06 (0.94-1.18) | 1.27 (1.14-1.42) | 1.27 (1.14-1.42) | 1.38 (1.23-1.54) | <0.001 | 1.21 (1.14-1.29) |
| Endometrial cancer, N (%) | 61 (0.2) | 56 (0.2) | 96 (0.3) | 139 (0.5) | 173 (0.6) |  |  |
| Fully-adjusted model | ref | 0.83 (0.58-1.19) | 1.36 (0.98-1.88) | 1.80 (1.32-2.44) | 1.98 (1.46-2.68) | <0.001 | 1.60 (1.37-1.87) |
| Ovarian cancer, N (%) | 75 (0.3) | 72 (0.2) | 55 (0.2) | 66 (0.2) | 79 (0.3) |  |  |
| Fully-adjusted model | ref | 0.97 (0.70-1.34) | 0.76 (0.53-1.07) | 0.93 (0.66-1.30) | 1.15 (0.83-1.61) | 0.203 | 1.13 (0.93-1.38) |
| Non-cancer events, N (%) | 1,241 (4.2) | 1,288 (4.4) | 1,322 (4.5) | 1,456 (4.9) | 1,809 (6.1) |  |  |
| Fully-adjusted model | ref | 0.99 (0.92-1.07) | 0.99 (0.92-1.08) | 1.05 (0.97-1.13) | 1.19 (1.11-1.29) | <0.001 | 1.12 (1.08-1.17) |
| CHF, N (%) | 53 (0.2) | 42 (0.1) | 62 (0.2) | 57 (0.2) | 67 (0.2) |  |  |
| Fully-adjusted model | ref | 0.68 (0.45-1.02) | 0.92 (0.63-1.33) | 0.75 (0.51-1.09) | 0.72 (0.49-1.05) | 0.225 | 0.87 (0.70-1.09) |
| COPD, N (%) | 372 (1.3) | 365 (1.2) | 343 (1.2) | 321 (1.1) | 317 (1.1) |  |  |
| Fully-adjusted model | ref | 0.95 (0.82-1.10) | 0.91 (0.78-1.05) | 0.82 (0.70-0.96) | 0.75 (0.64-0.88) | <0.001 | 0.85 (0.78-0.93) |
| MI, N (%) | 561 (1.9) | 580 (2.0) | 577 (2.0) | 594 (2.0) | 664 (2.2) |  |  |
| Fully-adjusted model | ref | 1.01 (0.90-1.14) | 1.00 (0.89-1.13) | 1.02 (0.90-1.15) | 1.10 (0.98-1.24) | 0.202 | 1.04 (0.98-1.12) |
| Dementia, N (%) | 99 (0.3) | 89 (0.3) | 74 (0.3) | 90 (0.3) | 95 (0.3) |  |  |
| Fully-adjusted model | ref | 0.92 (0.69-1.23) | 0.79 (0.58-1.07) | 0.98 (0.73-1.31) | 1.10 (0.82-1.49) | 0.906 | 1.01 (0.85-1.20) |
| Diabetes, N (%) | 205 (0.7) | 265 (0.9) | 331 (1.1) | 472 (1.6) | 802 (2.7) |  |  |
| Fully-adjusted model | ref | 1.14 (0.95-1.36) | 1.31 (1.10-1.56) | 1.61 (1.37-1.90) | 2.14 (1.82-2.51) | <0.001 | 1.71 (1.58-1.85) |
| Stroke, N (%) | 189 (0.6) | 197 (0.7) | 191 (0.6) | 210 (0.7) | 204 (0.7) |  |  |
| Fully-adjusted model | ref | 1.04 (0.85-1.28) | 1.03 (0.84-1.26) | 1.14 (0.94-1.40) | 1.12 (0.91-1.39) | 0.349 | 1.06 (0.94-1.18) |

Quintile cutoff points were 0.67, 0.90, 1.15, 1.48 nmol/L for TT and 7.50, 10.63, 14.30, 19.94 pmol/L for FT in women. The HRs of each group were compared with those in the bottom quintiles. Non-cancer events consisted of CHF, MI, COPD, stroke, dementia and diabetes. Fully-adjusted model was as mentioned in Methods. ICD10 of cancer: colorectal cancer, C18-20; lung cancer, C33-34; breast cancer, C50; endometrial cancer, .C54-55; ovarian cancer, C56. Abbreviations: HR, hazard ratio; CI, confidence interval; N, number; ref, reference; SD, standard deviation; ref, reference; CHF, congestive heart failure; COPD, chronic obstructive pulmonary disease; MI, myocardial infarction.

| **Supplementary Table 7: Stratified analysis of associations between total testosterone, free testosterone and risk of health span termination in men** | | | | | | | |
| --- | --- | --- | --- | --- | --- | --- | --- |
| **Subgroups** | **Total N** | **Total testosterone** | | | **Free testosterone** | | |
|  |  | **HR (95%CI)** | ***P* value** | ***P*-het** | **HR (95%CI)** | ***P* value** | ***P*-het** |
| Age (years) |  |  |  |  |  |  |  |
| < 60 | 86,901 | 0.95 (0.88-1.02) | 0.176 | 0.861 | 1.00 (0.94-1.07) | 0.973 | 0.729 |
| ≥ 60 | 58,580 | 0.96 (0.90-1.02) | 0.154 |  | 0.99 (0.93-1.04) | 0.622 |  |
| BMI (kg/m2) |  |  |  |  |  |  |  |
| < 30 | 112,164 | 0.96 (0.91-1.01) | 0.106 | 0.373 | 1.02 (0.97-1.07) | 0.441 | 0.161 |
| ≥ 30 | 33,317 | 1.01 (0.91-1.11) | 0.890 |  | 0.95 (0.88-1.03) | 0.240 |  |
| Townsend index |  |  |  |  |  |  |  |
| < median | 72,644 | 1.03 (0.96-1.10) | 0.388 | 0.006 | 1.05 (0.99-1.11) | 0.136 | 0.018 |
| ≥ median | 72,837 | 0.90 (0.85-0.96) | 0.002 |  | 0.95 (0.90-1.00) | 0.063 |  |
| College or university degree | |  |  |  |  |  |  |
| Yes | 51,064 | 0.98 (0.90-1.07) | 0.672 | 0.571 | 1.04 (0.97-1.12) | 0.273 | 0.135 |
| No | 93,204 | 0.95 (0.90-1.01) | 0.082 |  | 0.97 (0.93-1.02) | 0.288 |  |
| Smoking status |  |  |  |  |  |  |  |
| Never | 74,055 | 1.01 (0.94-1.09) | 0.688 | 0.055 | 1.05 (0.99-1.12) | 0.126 | 0.022 |
| Current & Previous | 70,997 | 0.93 (0.87-0.98) | 0.012 |  | 0.95 (0.90-1.01) | 0.085 |  |
| Alcohol intake |  |  |  |  |  |  |  |
| Heavy | 79,024 | 1.03 (0.97-1.10) | 0.309 | 0.001 | 1.03 (0.98-1.09) | 0.289 | 0.067 |
| Light & Moderate | 66,353 | 0.88 (0.83-0.95) | <0.001 |  | 0.95 (0.90-1.01) | 0.130 |  |
| Physical activity |  |  |  |  |  |  |  |
| High | 54,649 | 1.02 (0.94-1.10) | 0.615 | 0.109 | 1.03 (0.97-1.11) | 0.324 | 0.427 |
| Low & Moderate | 69,642 | 0.94 (0.88-1.00) | 0.062 |  | 1.00 (0.94-1.06) | 0.942 |  |
| Healthy diet |  |  |  |  |  |  |  |
| Yes | 96,263 | 0.95 (0.90-1.00) | 0.073 | 0.501 | 0.99 (0.94-1.04) | 0.563 | 0.456 |
| No | 47,564 | 0.98 (0.91-1.06) | 0.659 |  | 1.02 (0.95-1.09) | 0.616 |  |
| Family history of cancer | |  |  |  |  |  |  |
| Yes | 50,831 | 0.98 (0.91-1.06) | 0.655 | 0.635 | 1.00 (0.94-1.07) | 0.979 | 0.901 |
| No | 84,992 | 0.96 (0.90-1.02) | 0.207 |  | 1.00 (0.95-1.06) | 0.871 |  |
| Family history of CCVD | |  |  |  |  |  |  |
| Yes | 76,566 | 0.98 (0.92-1.04) | 0.530 | 0.504 | 1.02 (0.97-1.07) | 0.497 | 0.344 |
| No | 61,090 | 0.95 (0.88-1.02) | 0.172 |  | 0.98 (0.91-1.04) | 0.504 |  |
| Use of aspirin/ibuprofen | |  |  |  |  |  |  |
| Yes | 32,779 | 1.02 (0.94-1.12) | 0.605 | 0.126 | 1.00 (0.92-1.08) | 0.979 | 0.801 |
| No | 111,067 | 0.94 (0.89-1.00) | 0.034 |  | 0.99 (0.94-1.04) | 0.650 |  |

Stratified analysis was conducted with quintile 5 vs. quintile 1. Fully-adjusted model was as mentioned in **Methods**. Abbreviations: CI, confidence interval; HR, hazard ratio; *P*-het, *P* value of heterogeneity; BMI, body mass index; CCVD, cardiac-cerebral vascular disease.

| **Supplementary Table 8: Stratified analysis of associations between total testosterone, free testosterone and risk of health span termination in women** | | | | | | | | |
| --- | --- | --- | --- | --- | --- | --- | --- | --- |
| **Subgroups** | **Total N** | **Total testosterone** | | | | **Free testosterone** | | |
|  |  | **HR (95%CI)** | **P value** | **P-het** | | **HR (95%CI)** | **P value** | **P-het** |
| Age (years) |  |  |  |  | |  |  |  |
| < 60 | 91,278 | 1.09 (1.01-1.16) | 0.017 | 0.796 | | 1.13 (1.05-1.21) | 0.001 | 0.892 |
| ≥ 60 | 56,455 | 1.07 (1.01-1.14) | 0.022 |  | | 1.12 (1.05-1.19) | <0.001 |  |
| BMI (kg/m2) |  |  |  |  | |  |  |  |
| < 30 | 114,812 | 1.09 (1.03-1.15) | 0.002 | 0.913 | | 1.15 (1.08-1.21) | <0.001 | 0.498 |
| ≥ 30 | 32,921 | 1.08 (0.99-1.18) | 0.065 |  | | 1.19 (1.07-1.33) | 0.001 |  |
| Townsend index |  |  |  |  | |  |  |  |
| < median | 73,780 | 1.10 (1.03-1.18) | 0.004 | 0.528 | | 1.11 (1.04-1.19) | 0.002 | 0.668 |
| ≥ median | 73,953 | 1.07 (1.00-1.14) | 0.035 |  | | 1.14 (1.06-1.21) | <0.001 |  |
| College or university degree | |  |  |  | |  |  |  |
| Yes | 47,790 | 1.08 (0.99-1.18) | 0.094 | 0.841 | | 1.09 (1.00-1.19) | 0.064 | 0.469 |
| No | 98,749 | 1.09 (1.03-1.15) | 0.002 |  | | 1.13 (1.07-1.20) | <0.001 |  |
| Smoking status |  |  |  |  | |  |  |  |
| Never | 88,968 | 1.08 (1.02-1.15) | 0.012 | 0.883 | | 1.12 (1.05-1.20) | <0.001 | 0.969 |
| Current & Previous | 58,308 | 1.09 (1.02-1.16) | 0.013 |  | | 1.12 (1.05-1.20) | 0.001 |  |
| Alcohol intake |  |  |  |  | |  |  |  |
| Heavy | 58,298 | 1.14 (1.06-1.23) | 0.000 | 0.079 | | 1.18 (1.09-1.28) | <0.001 | 0.126 |
| Light & Moderate | 89,347 | 1.05 (0.99-1.11) | 0.082 |  | | 1.09 (1.03-1.16) | 0.003 |  |
| Physical activity |  |  |  |  | |  |  |  |
| High | 45,063 | 1.11 (1.02-1.21) | 0.014 | 0.708 | | 1.15 (1.06-1.26) | 0.001 | 0.880 |
| Low & Moderate | 71,180 | 1.09 (1.02-1.17) | 0.010 |  | | 1.14 (1.07-1.23) | <0.001 |  |
| Healthy diet |  |  |  |  | |  |  |  |
| Yes | 121,119 | 1.08 (1.03-1.14) | 0.003 | 0.808 | | 1.13 (1.07-1.19) | <0.001 | 0.734 |
| No | 25,706 | 1.10 (0.98-1.22) | 0.094 |  | | 1.10 (0.99-1.23) | 0.081 |  |
| Family history of cancer | |  |  |  | |  |  |  |
| Yes | 52,132 | 1.10 (1.02-1.18) | 0.012 | 0.969 | | 1.13 (1.05-1.22) | 0.001 | 0.981 |
| No | 88,789 | 1.10 (1.03-1.16) | 0.003 |  | | 1.13 (1.06-1.21) | <0.001 |  |
| Family history of CCVD | |  |  |  | |  |  |  |
| Yes | 85,311 | 1.12 (1.05-1.18) | <0.001 | 0.220 | | 1.19 (1.12-1.27) | <0.001 | 0.004 |
| No | 57,197 | 1.05 (0.97-1.14) | 0.200 |  | | 1.03 (0.95-1.12) | 0.474 |  |
| Use of aspirin/ibuprofen | |  |  |  | |  |  |  |
| Yes | 35,182 | 1.02 (0.93-1.11) | 0.720 | 0.092 | | 1.09 (0.99-1.20) | 0.069 | 0.427 |
| No | 111,271 | 1.11 (1.06-1.17) | <0.001 |  | | 1.14 (1.08-1.20) | <0.001 |  |
| Ever had menopause |  |  |  |  | |  |  |  |
| Yes | 86,661 | 1.09 (1.03-1.15) | 0.002 | 0.259 | | 1.11 (1.05-1.18) | <0.001 | 0.527 |
| No | 40,142 | 1.01 (0.89-1.14) | 0.905 |  | | 1.06 (0.94-1.20) | 0.318 |  |
| Use of HRT |  |  |  |  | |  |  |  |
| Yes | 51,448 | 1.06 (0.99-1.13) | 0.102 | | 0.347 | 1.09 (1.02-1.17) | 0.011 | 0.415 |
| No | 95,908 | 1.11 (1.04-1.18) | 0.002 | |  | 1.14 (1.07-1.21) | <0.001 |  |

Stratified analysis was conducted with quintile 5 vs. quintile 1. Fully-adjusted model was as mentioned in **Methods**. Abbreviations: CI, confidence interval; HR, hazard ratio; P-het, p value of heterogeneity; BMI, body mass index; CCVD, cardiac-cerebral vascular disease; HRT, hormone replacement therapy.

| **Supplementary Table 9. Associations of total or free testosterone with incident health span termination with adjustment for PRS in men** | | | | | | |
| --- | --- | --- | --- | --- | --- | --- |
| **Exposure** | **No. of events (%)** | **Fully-adjusted model ^a^** | | **Fully-adjusted model +PRS** | | ***P* for Interaction** |
|  |  | **HR (95%CI)** | ***P* value** | **HR (95%CI)** | ***P* value** |  |
| **Total testosterone** |  |  |  |  |  | 0.207 |
| Quintile 1 | 5,940 (20.6) | 1.00 (ref) | ref | 1.00 (ref) | ref |  |
| Quintile 2 | 5,304 (18.4) | 0.94 (0.90-0.97) | 0.001 | 0.94 (0.90-0.98) | 0.001 |  |
| Quintile 3 | 5,210 (18.1) | 0.95 (0.91-0.98) | 0.007 | 0.95 (0.91-0.99) | 0.007 |  |
| Quintile 4 | 5,031 (17.5) | 0.94 (0.90-0.98) | 0.002 | 0.94 (0.90-0.98) | 0.002 |  |
| Quintile 5 | 5,020 (17.4) | 0.96 (0.92-1.00) | 0.075 | 0.96 (0.92-1.00) | 0.070 |  |
| **Free testosterone** |  |  |  |  |  | 0.585 |
| Quintile 1 | 6,705 (23.3) | 1.00 (ref) | ref | 1.00 (ref) | ref |  |
| Quintile 2 | 5,827 (20.2) | 0.98 (0.94-1.01) | 0.219 | 0.98 (0.94-1.01) | 0.212 |  |
| Quintile 3 | 5,218 (18.1) | 0.97 (0.94-1.01) | 0.108 | 0.97 (0.94-1.01) | 0.100 |  |
| Quintile 4 | 4,737 (16.4) | 0.99 (0.95-1.02) | 0.477 | 0.99 (0.95-1.02) | 0.444 |  |
| Quintile 5 | 4,018 (13.9) | 0.99 (0.96-1.04) | 0.805 | 0.99 (0.95-1.04) | 0.767 |  |

The HRs of each group were compared with those in the bottom quintiles. ^a^: Fully-adjusted model included age, ethnicity, college or university degree, deprivation index, body mass index, smoking status, alcohol drinking, IPAQ group, healthy diet, family history of CCVD or cancer, use of aspirin/ibuprofen PC1-10 and genotyping chip. SHBG was additionally adjusted in total testosterone. Abbreviations: CI, confidence interval; HR, hazard ratio; PRS, polygenetic risk score; ref, reference.

| **Supplementary Table 10. Associations of total or free testosterone with incident health span termination with adjustment for PRS in women** | | | | | | |
| --- | --- | --- | --- | --- | --- | --- |
| **Exposure** | **No. of events (%)** | **Fully-adjusted model ^a^** | | **Fully-adjusted model +PRS** | | ***P* for Interaction** |
|  |  | **HR (95%CI)** | ***P* value** | **HR (95%CI)** | ***P* value** |  |
| **Total testosterone** |  |  |  |  |  | 0.573 |
| Quintile 1 | 3,892 (13.3) | 1.00 (ref) | ref | 1.00 (ref) | ref |  |
| Quintile 2 | 3,828 (13.1) | 1.02 (0.98-1.07) | 0.284 | 1.02 (0.98-1.07) | 0.280 |  |
| Quintile 3 | 3,702 (12.7) | 1.02 (0.97-1.07) | 0.402 | 1.02 (0.97-1.07) | 0.423 |  |
| Quintile 4 | 3,577 (12.2) | 1.01 (0.96-1.06) | 0.712 | 1.01 (0.96-1.06) | 0.739 |  |
| Quintile 5 | 3,770 (12.9) | 1.09 (1.04-1.14) | <0.001 | 1.08 (1.04-1.14) | <0.001 |  |
| **Free testosterone** |  |  |  |  |  | 0.457 |
| Quintile 1 | 3,584 (12.3) | 1.00 (ref) | ref | 1.00 (ref) | ref |  |
| Quintile 2 | 3,589 (12.3) | 0.99 (0.94-1.04) | 0.657 | 0.99 (0.94-1.04) | 0.618 |  |
| Quintile 3 | 3,638 (12.5) | 1.01 (0.96-1.05) | 0.783 | 1.00 (0.96-1.06) | 0.849 |  |
| Quintile 4 | 3,800 (13.0) | 1.04 (1.00-1.09) | 0.069 | 1.04 (0.99-1.09) | 0.081 |  |
| Quintile 5 | 4,158 (14.2) | 1.12 (1.07-1.18) | <0.001 | 1.12 (1.07-1.17) | <0.001 |  |

The HRs of each group were compared with those in the bottom quintiles. ^a^: Fully-adjusted model included age, menopause, ethnicity, college or university degree, deprivation index, body mass index, smoking status, alcohol drinking, IPAQ group, healthy diet, family history of CCVD or cancer, use of aspirin/ibuprofen or HRT, PC1-10 and genotyping chip. SHBG was additionally adjusted in total testosterone. Abbreviations: CI, confidence interval; HR, hazard ratio; PRS, polygenetic risk score; ref, reference. HRT, hormone replacement therapy.

| **Supplementary Table 11. Sensitivity analyses excluding participants with health span end within the first two years of follow-up (*n*=5,749 in men, *n*=4,100 in women) for the associations between** **total testosterone, free testosterone and HST risk** | | | | | | | |
| --- | --- | --- | --- | --- | --- | --- | --- |
| **Exposure** | **Hazard Ratio (95% CI)** | | | | | ***P* for trend** | **HR per log SD increase** |
|  | **Quintile 1** | **Quintile 2** | **Quintile 3** | **Quintile 4** | **Quintile 5** |  |  |
| **Men (*n*=139,732)** |  |  |  |  |  |  |  |
| **Total testosterone** |  |  |  |  |  |  |  |
| No. of events (%) | 4,742 (17.0) | 4,231 (15.1) | 4,114 (14.7) | 3,953 (14.1) | 3,959 (14.2) |  |  |
| Fully-adjusted model | ref | 0.95 (0.91-0.99) | 0.94 (0.90-0.98) | 0.93 (0.89-0.97) | 0.96 (0.91-1.01) | 0.009 | 0.93 (0.88-0.98) |
| **Free testosterone** |  |  |  |  |  |  |  |
| No. of events (%) | 5,278 (18.9) | 4,638 (16.6) | 4,155 (14.9) | 3,747 (13.4) | 3,181 (11.4) |  |  |
| Fully-adjusted model | ref | 0.99 (0.96-1.03) | 0.99 (0.95-1.03) | 0.99 (0.95-1.04) | 1.00 (0.96-1.05) | 0.441 | 0.98 (0.93-1.03) |
| **Women (*n*=143,633)** |  |  |  |  |  |  |  |
| **Total testosterone** |  |  |  |  |  |  |  |
| No. of events (%) | 3,079 (10.7) | 3,039 (10.6) | 2,912 (10.1) | 2,834 (9.9) | 2,999 (10.4) |  |  |
| Fully-adjusted model | ref | 1.02 (0.97-1.08) | 1.01 (0.96-1.07) | 1.01 (0.96-1.07) | 1.09 (1.04-1.15) | 0.004 | 1.05 (1.02-1.09) |
| **Free testosterone** |  |  |  |  |  |  |  |
| No. of events (%) | 2,817 (9.8) | 2,828 (9.8) | 2,879 (10.0) | 3,016 (10.5) | 3,323 (11.6) |  |  |
| Fully-adjusted model | ref | 0.99 (0.94-1.05) | 1.01 (0.96-1.07) | 1.05 (0.99-1.11) | 1.13 (1.07-1.19) | <0.001 | 1.09 (1.06-1.12) |

The HRs of each group were compared with those in the bottom quintiles. Fully-adjusted model: age, ethnicity, menopause status(women), college or university degree, deprivation index, body mass index, smoking status, alcohol drinking, IPAQ group, healthy diet, family history of CCVD or cancer, use of aspirin/ibuprofen and HRT (women); SHBG was further adjusted in total testosterone. Abbreviations: CI, confidence interval; HR, hazard ratio; SD, standard deviation; ref, reference; CCVD, cardiac-cerebral vascular disease; IPAQ, international physical activity questionnaire; HRT, hormone replacement therapy

| **Supplementary Table 12. Sensitivity analyses excluding participants with self-reported poor health status at baseline (*n*=4,164 in men, *n*=3,264 in women) for the associations between total testosterone, free testosterone and HST risk** | | | | | | | |
| --- | --- | --- | --- | --- | --- | --- | --- |
| **Exposure** | **Hazard Ratio (95% CI)** | | | | | ***P* for trend** | **HR per log SD increase** |
|  | **Quintile 1** | **Quintile 2** | **Quintile 3** | **Quintile 4** | **Quintile 5** |  |  |
| **Men (*n*=141,317)** |  |  |  |  |  |  |  |
| **Total testosterone** |  |  |  |  |  |  |  |
| No. of events (%) | 5,643 (20.0) | 5,095 (18.0) | 5,037 (17.8) | 4,859 (17.2) | 4,770 (16.9) |  |  |
| Fully-adjusted model | ref | 0.95 (0.92-0.99) | 0.97 (0.93-1.01) | 0.96 (0.92-1.00) | 0.97 (0.93-1.02) | 0.017 | 0.94 (0.90-0.99) |
| **Free testosterone** |  |  |  |  |  |  |  |
| No. of events (%) | 6,364 (22.5) | 5,601 (19.8) | 5,029 (17.8) | 4,572 (16.2) | 3,838 (13.6) |  |  |
| Fully-adjusted model | ref | 0.99 (0.95-1.03) | 0.99 (0.95-1.03) | 1.01 (0.97-1.05) | 1.01 (0.97-1.05) | 0.594 | 0.99 (0.94-1.03) |
| **Women (*n*=144,469)** |  |  |  |  |  |  |  |
| **Total testosterone** |  |  |  |  |  |  |  |
| No. of events (%) | 3,751 (13.0) | 3,729 (12.9) | 3,579 (12.4) | 3,459 (12.0) | 3,655 (12.6) |  |  |
| Fully-adjusted model | ref | 1.03 (0.99-1.08) | 1.03 (0.98-1.08) | 1.02 (0.97-1.07) | 1.10 (1.05-1.15) | <0.001 | 1.06 (1.03-1.10) |
| **Free testosterone** |  |  |  |  |  |  |  |
| No. of events (%) | 3,459 (12.0) | 3,498 (12.1) | 3,527 (12.2) | 3,689 (12.8) | 4,000 (13.8) |  |  |
| Fully-adjusted model | ref | 1.00 (0.96-1.05) | 1.02 (0.97-1.07) | 1.06 (1.01-1.11) | 1.14 (1.09-1.19) | <0.001 | 1.09 (1.06-1.12) |

The HRs of each group were compared with those in the bottom quintiles. Fully-adjusted model: age, ethnicity, menopause status(women), college or university degree, deprivation index, body mass index, smoking status, alcohol drinking, IPAQ group, healthy diet, family history of CCVD or cancer, use of aspirin/ibuprofen and HRT (women); SHBG was further adjusted in total testosterone. Abbreviations: CI, confidence interval; HR, hazard ratio; SD, standard deviation; ref, reference; CCVD, cardiac-cerebral vascular disease; IPAQ, international physical activity questionnaire; HRT, hormone replacement therapy.

| **Supplementary Table 13. Sensitivity analyses excluding participants with outliers for the associations between total testosterone, free testosterone and HST risk** | | | | | | | |
| --- | --- | --- | --- | --- | --- | --- | --- |
| **Exposure** | **Hazard Ratio (95% CI)** | | | | | ***P*** for trend | **HR per log SD increase** |
|  | **Quintile 1** | **Quintile 2** | **Quintile 3** | **Quintile 4** | **Quintile 5** |  |  |
| **Men** |  |  |  |  |  |  |  |
| **Total** **testosterone (*n*=142,568)** |  |  |  |  |  |  |  |
| No. of events (%) | 5,780 (20.3) | 5,234 (18.4) | 5,161 (18.1) | 4,994 (17.5) | 4,941 (17.3) |  |  |
| Fully-adjusted model | ref | 0.95 (0.92-0.99) | 0.96 (0.93-1.00) | 0.95 (0.91-0.99) | 0.97 (0.93-1.02) | 0.028 | 0.94 (0.89-0.99) |
| **Free testosterone (*n*=142,571)** |  |  |  |  |  |  |  |
| No. of events (%) | 6,520 (22.9) | 5,756 (20.2) | 5,176 (18.2) | 4,708 (16.5) | 4,002 (14.0) |  |  |
| Fully-adjusted model | ref | 0.99 (0.96-1.03) | 0.99 (0.95-1.02) | 1.00 (0.97-1.04) | 1.01 (0.97-1.05) | 0.452 | 1.02 (0.97-1.07) |
| **Women** |  |  |  |  |  |  |  |
| **Total testosterone (*n*=144,769)** |  |  |  |  |  |  |  |
| No. of events (%) | 3,840 (13.3) | 3,773 (13.1) | 3,680 (12.7) | 3,532 (12.2) | 3,691 (12.7) |  |  |
| Fully-adjusted model | ref | 1.02 (0.98-1.07) | 1.03 (0.98-1.07) | 1.01 (0.97-1.06) | 1.08 (1.03-1.13) | 0.005 | 1.05 (1.01-1.08) |
| **Free testosterone (*n*=144,777)** |  |  |  |  |  |  |  |
| No. of events (%) | 3,582 (12.4) | 3,549 (12.3) | 3,604 (12.4) | 3,770 (13.0) | 4,045 (14.0) |  |  |
| Fully-adjusted model | ref | 0.98 (0.94-1.03) | 1.00 (0.96-1.05) | 1.04 (0.99-1.09) | 1.10 (1.05-1.15) | <0.001 | 1.07 (1.04-1.10) |

Top 1% and bottom 1% of total/free testosterone were considered as the outliers (Men: total testosterone, n=2913, free testosterone, n=2910; Women: total testosterone, n=2964, free testosterone, n=2956). The HRs of each group were compared with those in the bottom quintiles. Fully-adjusted model: age, ethnicity, menopause status(women), college or university degree, deprivation index, body mass index, smoking status, alcohol drinking, IPAQ group, healthy diet, family history of CCVD or cancer, use of aspirin/ibuprofen and HRT (women); SHBG was further adjusted in total testosterone. Abbreviations: CI, confidence interval; HR, hazard ratio; SD, standard deviation; ref, reference; CCVD, cardiac-cerebral vascular disease; IPAQ, international physical activity questionnaire; HRT, hormone replacement therapy.

| **Supplementary Table 14. Sensitivity analyses adjusting fasting time for the associations between total testosterone, free testosterone and HST risk** | | | | | | | |
| --- | --- | --- | --- | --- | --- | --- | --- |
| **Exposure** | **Hazard Ratio (95% CI)** | | | | | ***P* for trend** | **HR per log SD increase** |
|  | **Quintile 1** | **Quintile 2** | **Quintile 3** | **Quintile 4** | **Quintile 5** |  |  |
| **Men (*n*=145,481)** |  |  |  |  |  |  |  |
| **Total testosterone** |  |  |  |  |  |  |  |
| No. of events (%) | 5,988 (20.6) | 5,354 (18.4) | 5,255 (18.1) | 5,085 (17.5) | 5,066 (17.4) |  |  |
| Multivariable model 1 ^a^ | ref | 0.94 (0.91-0.98) | 0.95 (0.91-0.99) | 0.94 (0.90-0.98) | 0.96 (0.92-1.01) | <0.001 | 0.92 (0.88-0.97) |
| Multivariable model 2 ^b^ | ref | 0.94 (0.91-0.98) | 0.95 (0.91-0.98) | 0.94 (0.90-0.97) | 0.96 (0.91-1.00) | <0.001 | 0.92 (0.87-0.96) |
| **Free testosterone** |  |  |  |  |  |  |  |
| No. of events (%) | 6,761 (23.2) | 5,879 (20.2) | 5,279 (18.1) | 4,776 (16.4) | 4,053 (13.9) |  |  |
| Multivariable model 1 | ref | 0.98 (0.94-1.01) | 0.97 (0.94-1.01) | 0.99 (0.95-1.02) | 0.99 (0.95-1.03) | 0.150 | 0.97 (0.93-1.01) |
| Multivariable model 2 | ref | 0.98 (0.94-1.01) | 0.97 (0.94-1.01) | 0.98 (0.95-1.02) | 0.99 (0.95-1.03) | 0.100 | 0.96 (0.92-1.01) |
| **Women (*n*=147,733)** |  |  |  |  |  |  |  |
| **Total testosterone** |  |  |  |  |  |  |  |
| No. of events (%) | 3,936 (13.3) | 3,869 (13.1) | 3,742 (12.7) | 3,608 (12.2) | 3,808 (12.9) |  |  |
| Multivariable model 1 | ref | 1.02 (0.98-1.07) | 1.02 (0.97-1.07) | 1.01 (0.96-1.05) | 1.08 (1.04-1.13) | 0.001 | 1.05 (1.02-1.08) |
| Multivariable model 2 | ref | 1.02 (0.98-1.07) | 1.02 (0.97-1.07) | 1.01 (0.96-1.05) | 1.08 (1.03-1.13) | 0.002 | 1.05 (1.02-1.08) |
| **Free testosterone** |  |  |  |  |  |  |  |
| No. of events (%) | 3,621 (12.3) | 3,633 (12.3) | 3,675 (12.4) | 3,838 (13.0) | 4,196 (14.2) |  |  |
| Multivariable model 1 | ref | 0.99 (0.95-1.04) | 1.01 (0.96-1.06) | 1.04 (1.00-1.09) | 1.12 (1.07-1.18) | <0.001 | 1.08 (1.05-1.11) |
| Multivariable model 2 | ref | 0.99 (0.95-1.04) | 1.01 (0.96-1.06) | 1.04 (1.00-1.09) | 1.12 (1.07-1.18) | <0.001 | 1.08 (1.05-1.10) |

^a^ Multivariable model 1: age, ethnicity, menopause status(women), college or university degree, deprivation index, body mass index, smoking status, alcohol drinking, IPAQ group, healthy diet, family history of CCVD or cancer, use of aspirin/ibuprofen and HRT (women), SHBG was further adjusted in total testosterone; ^b^ Multivariable model 2: all the covariates were included in multivariable model 1 and fasting time (continuous, hour) as an additional covariate; The HRs of each group were compared with those in the bottom quintiles; Abbreviations: CI, confidence interval; HR, hazard ratio; SD, standard deviation; ref, reference; CCVD, cardiac-cerebral vascular disease; IPAQ, international physical activity questionnaire; HRT, hormone replacement therapy.

| **Supplementary Table 15. Sensitivity analyses adjusting menstrual cycle proxy factors for the associations between testosterone, free testosterone and health span termination risk in women (*n*=147,733)** | | | | | | | |
| --- | --- | --- | --- | --- | --- | --- | --- |
| **Exposure** | **Hazard Ratio (95% CI)** | | | | | ***P* for trend** | **HR per log SD increase** |
|  | **Quintile 1** | **Quintile 2** | **Quintile 3** | **Quintile 4** | **Quintile 5** |  |  |
| **Total testosterone** |  |  |  |  |  |  |  |
| No. of events (%) | 3,936 (13.3) | 3,869 (13.1) | 3,742 (12.7) | 3,608 (12.2) | 3,808 (12.9) |  |  |
| Multivariable model 1 ^a^ | ref | 1.02 (0.98-1.07) | 1.02 (0.97-1.07) | 1.01 (0.96-1.05) | 1.08 (1.04-1.13) | 0.001 | 1.05 (1.02-1.08) |
| Multivariable model 2 ^b^ | ref | 1.02 (0.98-1.07) | 1.02 (0.97-1.07) | 1.01 (0.96-1.05) | 1.08 (1.04-1.13) | 0.001 | 1.05 (1.02-1.08) |
| Multivariable model 3 ^c^ | ref | 1.02 (0.98-1.07) | 1.02 (0.97-1.07) | 1.01 (0.96-1.05) | 1.08 (1.04-1.13) | 0.001 | 1.05 (1.02-1.08) |
| Multivariable model 4 ^d^ | ref | 1.02 (0.98-1.07) | 1.02 (0.97-1.07) | 1.01 (0.96-1.05) | 1.08 (1.04-1.14) | 0.001 | 1.05 (1.02-1.08) |
| **Free testosterone** |  |  |  |  |  |  |  |
| No. of events (%) | 3,621 (12.3) | 3,633 (12.3) | 3,675 (12.4) | 3,838 (13.0) | 4,196 (14.2) |  |  |
| Multivariable model 1 | ref | 0.99 (0.95-1.04) | 1.01 (0.96-1.06) | 1.04 (1.00-1.09) | 1.12 (1.07-1.18) | <0.001 | 1.08 (1.05-1.11) |
| Multivariable model 2 | ref | 0.99 (0.95-1.04) | 1.01 (0.96-1.06) | 1.04 (1.00-1.09) | 1.12 (1.07-1.18) | <0.001 | 1.08 (1.05-1.11) |
| Multivariable model 3 | ref | 0.99 (0.95-1.04) | 1.01 (0.96-1.06) | 1.04 (1.00-1.09) | 1.12 (1.07-1.18) | <0.001 | 1.08 (1.05-1.11) |
| Multivariable model 4 | ref | 0.99 (0.95-1.04) | 1.01 (0.96-1.06) | 1.04 (1.00-1.09) | 1.12 (1.07-1.18) | <0.001 | 1.08 (1.05-1.11) |

Menstrual cycle proxy factors: time since last menstrual period (factor, 0-7 day, 8-14 day, 15-21 day, 22-28 day, 29-35 day, ≥36 day, menopause); length of menstrual cycle (factor, 7-21 day, 22-28 day, 29-35 day, irregular cycle, menopause); menstruating today (factor, no, yes, menopause); ^a^ Multivariable model 1: age, ethnicity, menopause status(women), college or university degree, deprivation index, body mass index, smoking status, alcohol drinking, IPAQ group, healthy diet, family history of CCVD or cancer, use of aspirin/ibuprofen and HRT (women), SHBG was further adjusted in total testosterone; ^b^ Multivariable model 2: multivariable model 1 + time since last menstrual period; ^c^ Multivariable model 3: multivariable model 1 + length of menstrual cycle; ^d^ Multivariable model 4: multivariable model 1 + menstruating today; The HRs of each group were compared with those in the bottom quintiles; Abbreviations: CI, confidence interval; HR, hazard ratio; SD, standard deviation; ref, reference; CCVD, cardiac-cerebral vascular disease; IPAQ, international physical activity questionnaire; HRT, hormone replacement therapy.

**Supplementary Figure 1. Study design and workflow.**


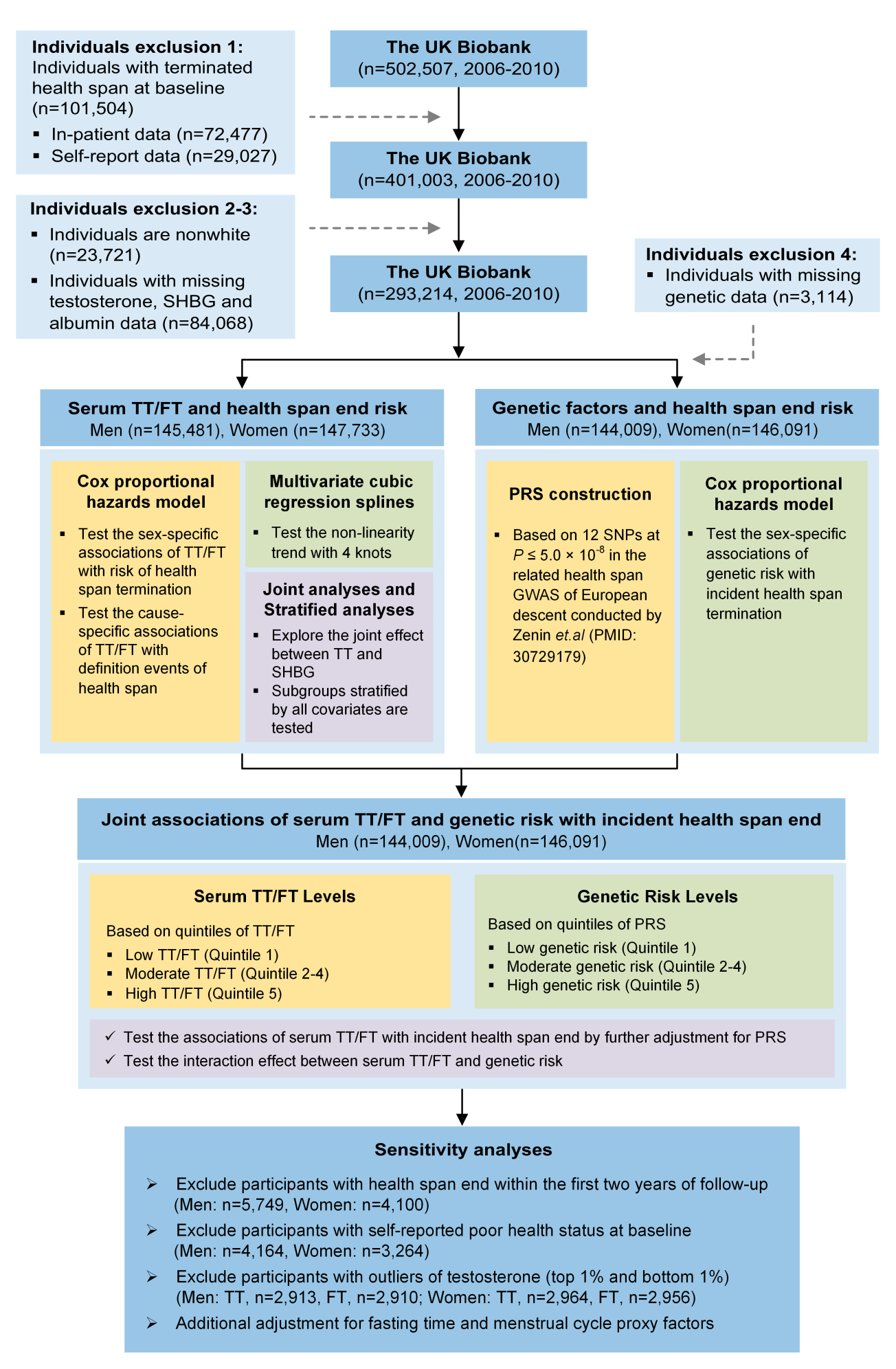


**Supplementary Figure 2. Distribution of total testosterone and free testosterone in men and women.**


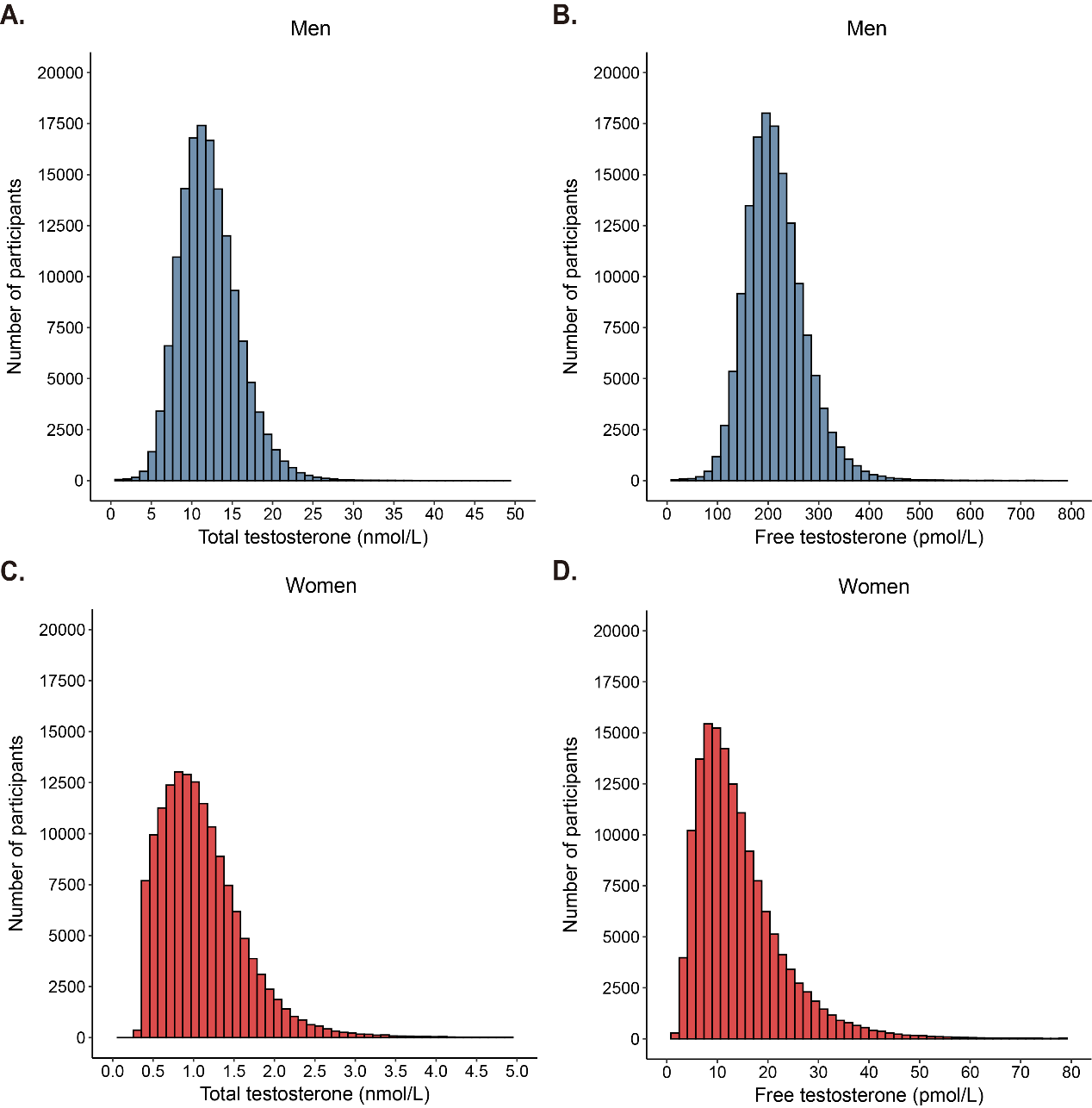


A) Total testosterone in men, B) Free testosterone in men, C) Total testosterone in women, D) Free testosterone in women. Blue represented the men and red represented the women. X-axis was the concentration of testosterone and Y-axis was the number of participants. The unit of total testosterone was 'nmol/L' and 'pmol/L' was for free testosterone. All of P for non-normality were <0.001.

**Supplementary Figure 3. Joint analyses of total testosterone and sex hormone binding globulin with health span in the fully-adjusted models.**

**
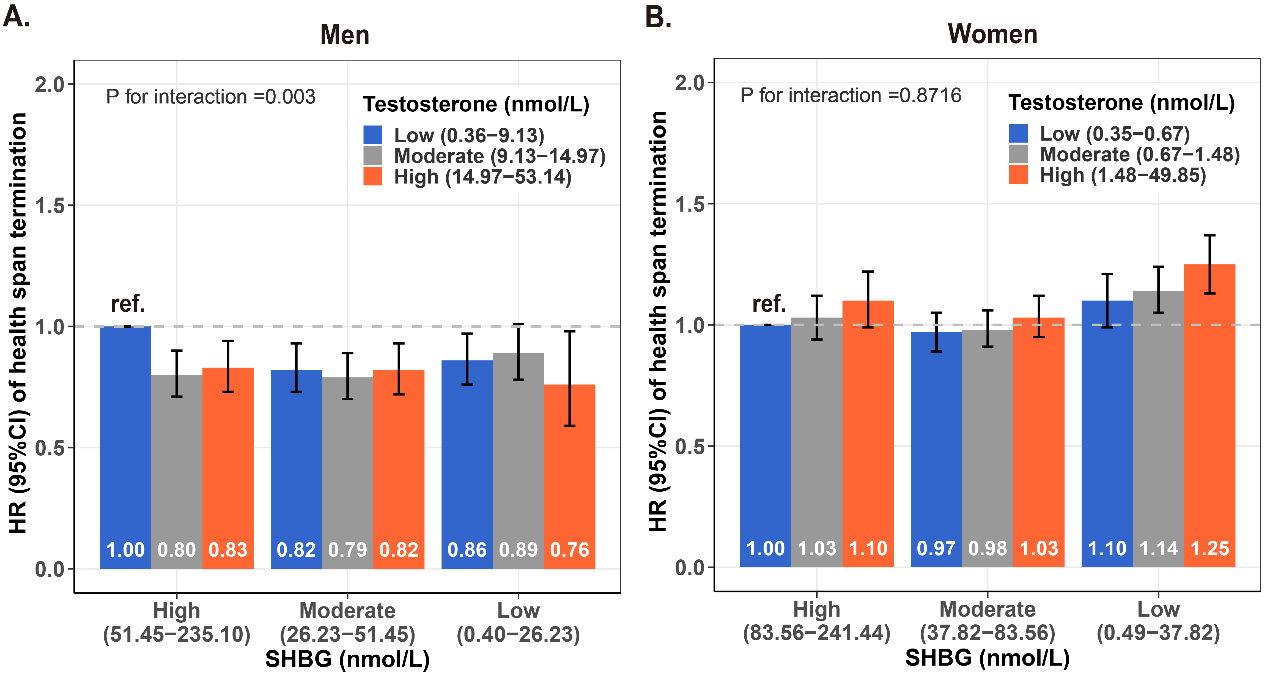
**

A) joint analyses of men, B) joint analyses of women. X-axis was the strata of sex hormone binding globulin and Y-axis was the hazard ratio of health span termination. Testosterone and SHBG were both divided into three strata: low (Quintile 1), moderate (Quintile 2-4) and high (Quintile 5). Participants both with low level of testosterone and high level of SHBG had the lowest level of free testosterone, thus this combination was considered as the reference in both genders. SHBG, sex hormone binding globulin.

**Supplementary Figure 4: Distribution of polygenetic risk score between terminated and unterminated health span participants.**


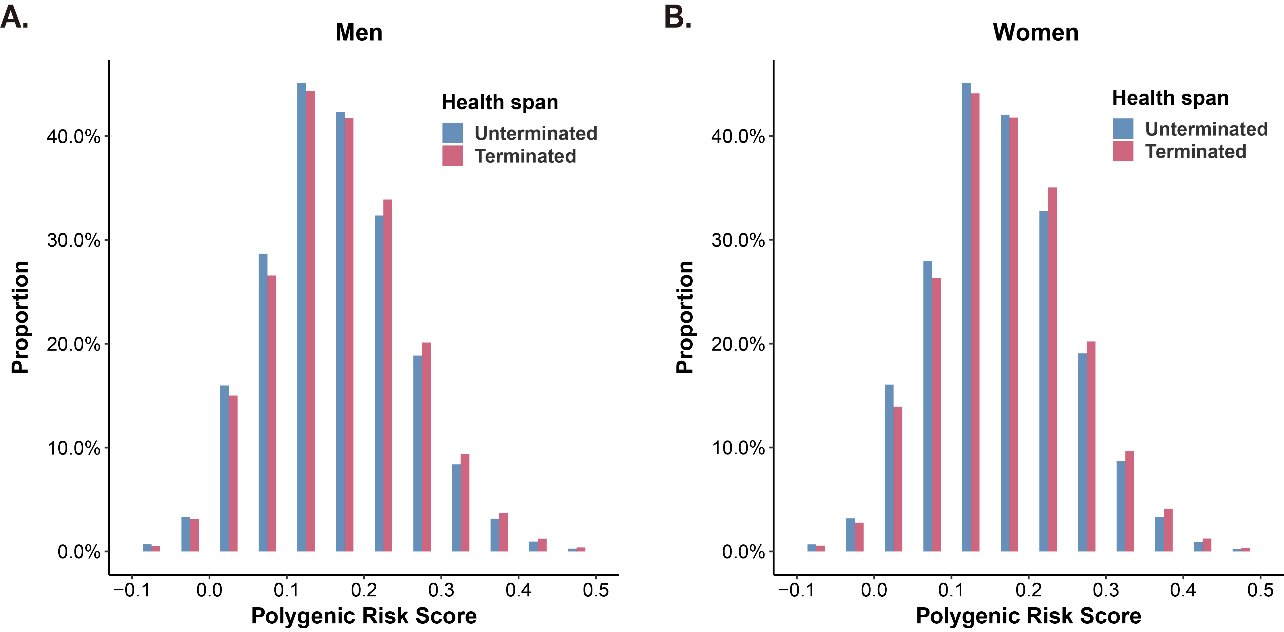


A) Distribution in men, B) distribution in women. X-axis was the polygenetic risk score and Y-axis was the proportion of participants. Blue represented the men and red represented the women.
